# Global Prevalence and Modifiers of Human Papillomavirus Positivity in Oral Cavity Cancer: A Systematic Review and Meta-Analysis of Prevalence (1995–2024)

**DOI:** 10.3390/cancers17172870

**Published:** 2025-08-31

**Authors:** Areeb Iraqui, Alaa Safia, Mohamad Mahameed, Uday Abd Elhadi, Shlomo Merchavy

**Affiliations:** Department of Otolaryngology, Head & Neck Surgery Unit, Ziv Medical Center, 1311001 Safed, Israel

**Keywords:** human papillomavirus, oral cavity cancer, HPV-16, HPV-18, prevalence, systematic review, meta-analysis

## Abstract

**Background/Objectives**: Human papillomavirus (HPV) is a known etiologic agent in oropharyngeal cancers, but its role in oral cavity squamous cell carcinoma (OCSCC) remains unclear. This systematic review and meta-analysis aimed to estimate the global prevalence of HPV in OCSCC and explore variation by clinicodemographic and tumor characteristics. **Methods:** We systematically searched multiple databases for studies reporting HPV prevalence in OCSCC. Pooled prevalence estimates were calculated, and subgroup analyses examined differences by age, gender, cancer stage, anatomical site, histologic subtype, region, and HPV type (HPV-16 and HPV-18). Heterogeneity and publication bias were assessed using standard meta-analytic techniques. **Results:** A total of 122 studies involving 16,311 patients were included. The pooled HPV prevalence in OCSCC was 25.8% (95% CI: 20.4–31.2), with HPV-16 and HPV-18 detected in 52.4% and 30.3% of positive cases, respectively. Prevalence varied geographically, from 73% in Singapore to 7.7% in South Korea. Younger patients (<40 years) had higher HPV positivity (29.7%) than older patients (>70 years, 23.8%). Early-stage cancers (stage I) showed higher HPV prevalence (41.8%) than advanced-stage cancers (stage IV, 10.4%). Verrucous carcinoma had the highest HPV positivity (34.1%), and moderately differentiated tumors the lowest (23.4%). HPV prevalence was highest in the lower alveolus (29.5%) and lips (25%), and lowest in the upper gingiva (3.9%). **Conclusions:** HPV prevalence in OCSCC demonstrates significant heterogeneity across regions and clinical subgroups. These findings emphasize the need for standardized diagnostic approaches and further research into the role of HPV in OCSCC pathogenesis and treatment.

## Simple Summary

Oral cavity cancer is a serious disease affecting thousands of people worldwide. While a virus called human papillomavirus (HPV) is known to play a major role in throat cancers, its role in oral cavity cancers is still unclear. We reviewed and analyzed data from over 16,000 patients across 122 studies to better understand how often HPV is found in oral cavity cancers and whether this depends on age, gender, tumor site, or region. We found that HPV is present in about one in four cases, but the rates vary widely across countries and patient groups. These findings suggest that HPV may play a role in some—but not all—oral cancers. Our study highlights the importance of further research and consistent testing methods to better understand how HPV affects cancer in the mouth and how this knowledge can improve prevention and treatment strategies.

## 1. Introduction

Human papillomavirus (HPV) is a well-recognized etiological agent in a variety of cancers, including cervical, anogenital, and head and neck cancers [1]. Among head and neck cancers, HPV’s role is well-established in oropharyngeal squamous cell carcinoma (OPSCC) [2], where its presence is associated with distinct clinical and biological characteristics, including improved prognosis and responsiveness to treatment. However, the contribution of HPV to oral cavity squamous cell carcinoma (OCSCC) remains less clear and continues to be a subject of scientific debate [3,4].

The global burden of oral cavity cancer is significant, accounting for approximately 300,000 new cases annually [5]. While traditional risk factors such as tobacco use, alcohol consumption, and poor oral hygiene predominate, recent evidence suggests a role for high-risk HPV subtypes, particularly HPV-16 and HPV-18, in the pathogenesis of OCSCC [6]. Unlike OPSCC, the prognostic and therapeutic implications of HPV positivity in OCSCC are inconsistent, with studies reporting conflicting associations between HPV status and clinical outcomes [7].

The existing literature highlights substantial variability in HPV prevalence across geographic regions, patient demographics, and tumor characteristics, reflecting differences in HPV detection methods and population exposures [8,9,10,11,12,13,14]. Notably, the prevalence of high-risk HPV subtypes in OCSCC is often lower than in OPSCC, raising questions about the biological significance of HPV in oral carcinogenesis [14,15,16,17]. Furthermore, the interplay between HPV and other etiological factors, such as smoking and alcohol use, remains poorly understood.

In this context, a comprehensive understanding of the prevalence of HPV and its subtypes in OCSCC is critical to refining prevention, diagnosis, and treatment strategies. This systematic review and meta-analysis aim to quantify the global prevalence of HPV in OCSCC and explore its variability based on clinicodemographic factors, including age, gender, cancer stage, tumor site, and histological type. By synthesizing data from a diverse array of studies, we seek to elucidate the role of HPV in OCSCC and its implications for clinical and public health practices.

## 2. Materials and Methods

### 2.1. Design and Literature Search

The study protocol of this systematic review was registered on PROSPERO (CRD42024615069). This work was conducted following the PRISMA [18] (Preferred Reporting Items for Systematic Reviews and Meta-Analyses) and AMSTAR [19] (Assessing the methodological quality of systematic reviews) guidelines. We searched PubMed, Scopus, Web of Science, the Cochrane Library, and Google Scholar (first 200 records) [20] up to 17 October 2024. The search strategy, outlined in Table S1, was adjusted for each database. Citations were filtered based on their titles and abstracts. No restrictions were applied regarding the original language of publication. Manual searches were conducted by reviewing reference lists and related articles on PubMed [21] and Google Scholar.

### 2.2. Selection Strategy

Studies were selected using the PECO (Population, Exposure, Comparison, and Outcomes) framework [22].

The inclusion criteria were as follows:

1. Population: patients with histologically confirmed oral cavity cancer.
2. Exposure: none.
3. Comparison: none.
4. Outcome: HPV-positive rate.
5. Study Design: epidemiological studies and cross-sectional studies. Case-control studies were only considered if they investigated the rate of HPV positivity in cancer and healthy individuals.

The exclusion criteria included the following:

1. Non-original research.
2. Abstract-only publications.
3. Experimental and investigation studies (clinical trials).
4. Case reports and case series.
5. Case-control studies including HPV-positive and HPV-negative controls.
6. Duplicated records or studies with overlapping datasets (similar samples and baseline characteristics even if author lists differed).
7. Non-oral cavity cancer (like oropharyngeal cancer—OPC).
8. Studies including patients with oral cavity cancer and OPC without stratifying HPV data based on cancer location.
9. Studies not reporting HPV-positivity rate.
10. Animal studies plus in vivo or in vitro studies.

### 2.3. Data Collection and Outcomes

The senior author designed the data collection sheet using Microsoft Excel. The sheet was modified multiple times to fit the data reported by the included studies. The final sheet comprised four parts. The first covered study-related data (authors’ names, year of publication/investigation, country of investigation, study design, and sample size). The second covered patient-related data (including age, gender, site of cancer, management type, and diagnostic method of both HPV and oral cavity cancer). The third part included the outcome data. The primary outcome was the prevalence of HPV positivity—overall and across various strains, specifically HPV-16 and -18 since they carry the highest risk. Exploratory/secondary analyses were conducted across various subsets of patients based on age group, gender, smoking status, alcohol intake, cancer site, management type, tumor stage (pathological and clinical), histopathological grading, and p16 immunohistochemistry (as a potential surrogate for HPV-positive cancers). A complete list of definitions used in this study can be found in the Supplementary Files. The fourth part included a methodological quality assessment.

To avoid double counting, we screened for potential cohort overlap across studies by cross-checking institution and location, recruitment years, author teams, and detection methods. When two reports could represent the same or partially overlapping series, we prioritized the report with the broader or more informative dataset and only retained a second report if it contributed an independent time window or unique subgroup data with its own denominator.

### 2.4. Risk of Bias Assessment

For all of the included studies, the National Institute of Health (NIH) tool was used. This tool assesses the methodological quality of observational studies through 14 questions, each of which can be given a score of 0, 1, or 2. This provides an overall quality score of good (score > 20), fair (score 11–20), and poor (score < 11). This scoring method has been previously employed widely and validated.

### 2.5. Statistical Analysis

All analyses were performed using STATA (Version 18, StataCorp LLC, College Station, TX, USA) following the a priori analysis plan. To account for the highly heterogeneous samples included in the quantitative synthesis, a random effects method was employed using the restricted maximum likelihood method (REML) to minimize the risk of missing data [23]. Heterogeneity was quantified using the I^2^ statistic, with significant heterogeneity defined as I^2^ > 40% [24].

Separate analyses were conducted for overall HPV and for specific HPV strains (HPV-11, -16, -18, -26, -33, -35, -52, -58, -65, and other strains). Since the HPV-16 and HPV-18 strains carry the most significant risks of cancer, they were analyzed and reported as secondary outcomes. For certain strains (HPV-2, -6, -16E, and -16Af-1/2) data were insufficient for analysis. Subgroup analyses were then performed to determine country-, year-, patient-, and cancer-specific changes in prevalence of HPV positivity.

Sensitivity analyses tested the robustness of results, with Galbraith plots identifying outliers, and publication bias was assessed with funnel plots and asymmetry tests [25]. No changes were observed with sensitivity analyses and no significant risks of publication bias were noted.

## 3. Results

### 3.1. Literature Search Results

**Figure 1:**
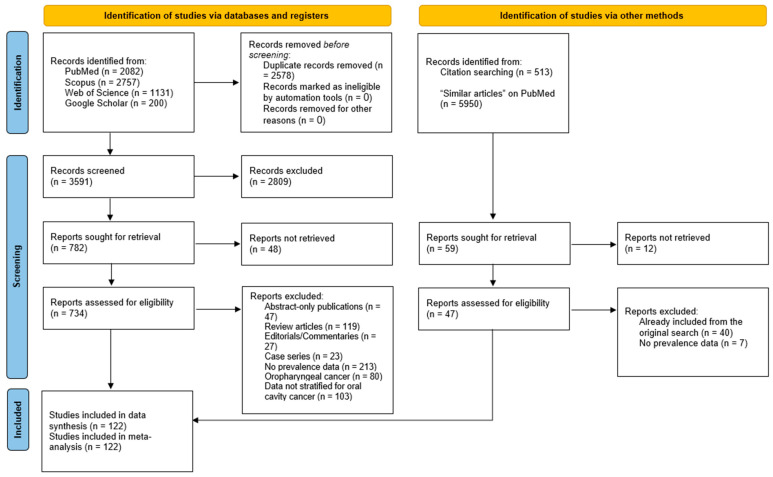
A PRISMA flow diagram showing the results of the literature search and screening process.

The systematic literature search identified 6170 records across multiple databases (Figure 1). After removing 2578 duplicate records, 3591 unique records were screened for eligibility. Following an initial title and abstract screening, 2809 records were ruled out. A total of 782 full-text reports were sought for retrieval, with 48 reports not accessible. Of the 734 full-text reports assessed for eligibility, 587 were excluded for various reasons, including lack of prevalence data (n = 213), focus on oropharyngeal rather than oral cavity cancer (n = 80), absence of stratified data for oral cavity cancer (n = 103), and publication formats not suitable for data extraction (e.g., abstract-only publications, review articles, editorials, and case series; n = 216). An additional 40 reports were excluded for overlap with the original search results. Ultimately, 122 studies met the inclusion criteria and were incorporated into the meta-analysis [8,9,10,11,13,14,15,16,17,26,27,28,29,30,31,32,33,34,35,36,37,38,39,40,41,42,43,44,45,46,47,48,49,50,51,52,53,54,55,56,57,58,59,60,61,62,63,64,65,66,67,68,69,70,71,72,73,74,75,76,77,78,79,80,81,82,83,84,85,86,87,88,89,90,91,92,93,94,95,96,97,98,99,100,101,102,103,104,105,106,107,108,109,110,111,112,113,114,115,116,117,118,119,120,121,122,123,124,125,126,127,128,129,130,131,132,133,134,135,136,137,138].

### 3.2. Baseline Characteristics

The included studies’ characteristics are summarized in Table 1. A total of 122 studies (97 cross-sectional and 25 case-control) investigated 16,311 patients with oral cancer, of whom 9528 (58.41%) males and 4177 (25.61%) females were examined. Overall HPV prevalence was reported in 120 (98.36%) of studies, with various subtypes being investigated as well, including HPV-16 (64, 52.45%), HPV-18 (37, 30.33%), HPV 11 (7, 5.74%), HPV-26 (3, 2.46%), HPV-33 (5, 4.09%), HPV-35 (3, 2.46%), HPV-52 (3, 2.46%), HPV-58 (8, 6.56%), and HPV-65 (1, 0.82%). Other strains were examined in 35 studies (28.69%); however, these strains were not classified and thus, were not investigated. Most evidence stemmed from India (24, 19.67%), followed by Thailand (7, 5.74%), Italy (6, 4.92%), and Japan (6, 4.92%). HPV-related data were available for various patients’ clinicodemographic data, including age (115 studies), gender (66 studies), smoking status (33 studies), alcohol intake (26 studies), cancer site (51 studies), TNM staging (25 studies), histological type (38 studies), p16 positivity (13 studies), and management type (29 studies). Data on specific cancer locations and HPV diagnosis can be found in Table 1.

### 3.3. Methodological Quality

A full description of the methodological quality of the included studies is provided in Table S2. Out of 122 studies, the majority had an overall fair methodological quality (92 studies, 75.41%), while the remaining 30 studies (24.59%) had an overall good quality, with no studies having a poor rating.

### 3.4. Country- and Year-Specific Prevalence Rates

**Figure 2:**
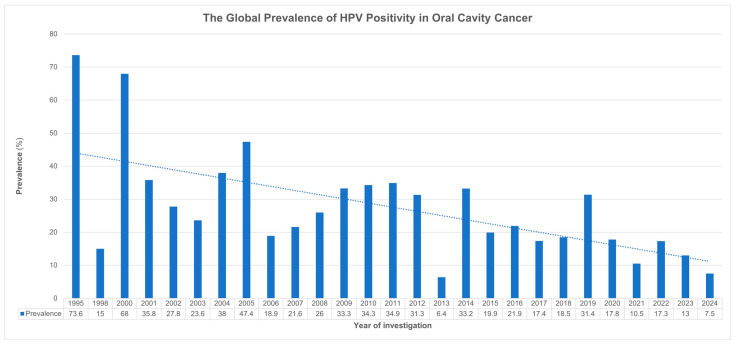
Trend analysis of HPV positivity in oral cavity cancer over time (1995–2024).

The overall positivity of HPV in oral cancer showed variable rates over time, with the highest rate being reported in 1995 (73.6%; 95% CI: 64.6–82.7) and the lowest rate in 2024 (7.5%; 95% CI: 1.9–13.1). A negative trend can be observed over time (Figure 2) despite the increasing body of evidence in later years (2014–2024) compared to earlier periods (1995–2014). Time was a significant moderator of HPV prevalence (*p* = 0.001), with heterogeneity measures being reported in Table S3.

**Figure 3:**
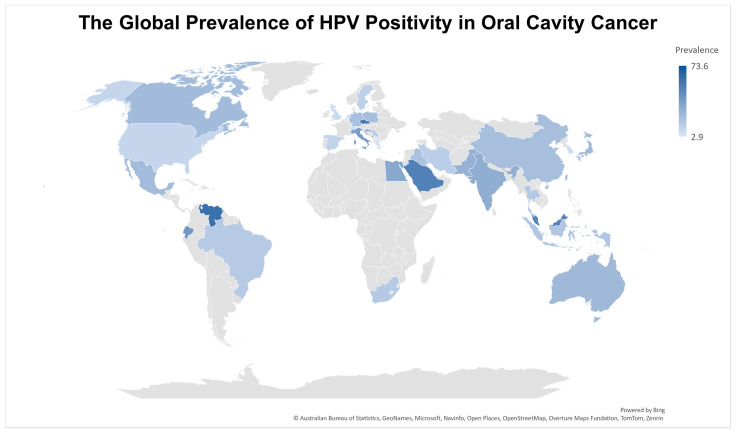
Country-specific prevalence of HPV positivity in oral cavity cancer.

Figure 3 shows the differences in HPV prevalence across various countries, with Singapore showing the highest rates (73%; 95% CI: 64.6–82.7) followed by Venezuela (60%; 95% CI: 46.4–73.6), the Czech Republic (55.7%, 95% CI: 46.5–65), Saudi Arabia (52.4%; 95% CI: 0–99.4), and Malaysia (51.4%; 95% CI: 41.9–61). Meanwhile, South Korea (7.7%; 95% CI: 3.3–10.6), Greece (5.4%, 95% CI: 0–15.3), and the Netherlands (4.3%, 95% CI: 0–9.4) had the lowest rates. Complete, country-based prevalence data can be found in Table S4.

### 3.5. Age- and Gender-Specific Prevalence

Age-specific prevalence rates show that HPV positivity tended to be highest in younger patients, with a steady reduction in rate as patients age. For instance, patients <40 years had a prevalence rate of 29.7% (95% CI: 20.3–39%) followed by 40–60 years (25.4%; 95% CI: 19.8–30.9%), 60–70 years (24.3%; 95% CI: 17.4–31.3%), and >70 years (23.8%; 95% CI: 8.6–39.1%) (Table 2). Meanwhile, female patients (24.6%; 95% CI: 19.3–29.8) had slightly higher, but non-significant, rates of HPV positivity compared to male patients (23.5%; 95% CI: 18.8–28.2, *p* = 0.059).

### 3.6. Smoking- and Alcohol-Specific Prevalence

The prevalence of HPV positivity in oral cancer was highest in current smokers, accounting for 27.2% (95% CI: 18.4–36%) of cases, comparable to that of those who never smoked (25.4%; 95% CI: 18.1–32.8%). Surprisingly, former smoking was associated with the lowest rate of 9.4% (95% CI: 0–19, I2 = 0%).

On the other hand, the rate of HPV positivity in oral cancer was similar across patients who reported ever (current plus past drinkers) (22.7%; 95% CI: 14.9–30.4) or never drinking alcohol (21.8%; 95% CI: 13.9–29.8). Strikingly, patients who reported excessive drinking habits (not defined) exhibited the lowest rates of HPV positivity (12.2%; 95% CI: 7.2–17.2); however, this finding was based only on a single observation.

### 3.7. Cancer Site-Specific Prevalence

Twenty-one oral cancer sites were examined, with the most frequently investigated sites being oral tongue (51 studies), buccal mucosa (39 studies), floor of mouth (38 studies), hard palate (24 studies), gingiva (20 studies), and lips (19 studies). Complete site-specific data can be found in Table 2. HPV positivity was highest in the lower alveolus (29.5%; 95% CI: 0–76.5%) followed by the lips (25%; 95% CI: 14.7–35.3%), mandibular (24.6%; 95% CI: 3.3–45.9) and maxillary gingiva (23.1%; 95% CI: 8.9–37.2), oral tongue (22.7%; 95% CI: 16.7–28.7), buccal mucosa (20.9%; 95% CI: 14.2–27.6), hard palate (18.9%; 95% CI: 10.8–26.9), and lower gingiva (18.8%; 95% CI: 2.3–35.3). Meanwhile, the gingivobuccal sulcus (4.7%; 95% CI: 0–13), upper gingiva (3.9%; 95% CI: 0–10.5), and vestibulum of mouth (0.4%; 95% CI: 0–1.5) exhibited the lowest rates.

### 3.8. Cancer Stage- and Grade-Specific Prevalence

Cancer staging was conducted using AJCC, pathological TNM, and clinical TNM staging systems. The prevalence of HPV positivity was highest in stage II (36.4%) followed by stage III (32.3%), stage I (31.9%), and stage IV (29.1%). Early-stage cancer (I–II) showed higher prevalence compared to advanced stages (III–IV) (28.8% vs. 27.7%).

These findings did not align with those of clinical TNM staging. For instance, the prevalence of HPV positivity was highest in the earlier stages with steady and progressive decline in advanced stages (stage I = 41.8%; stage II = 27.7%; stage III = 12.4%; stage IV = 10.4), with early stages having almost double the rate of advanced stages (I–II vs. III–IV = 24% vs. 12.7%).

Surprisingly, the prevalence rates were lower according to the AJCC staging system. For instance, the prevalence of HPV positivity in stages I to IV were 7.8%, 3.3%, 3.9%, and 10.3%, respectively.

In terms of histological grade, the highest prevalence of HPV was observed with verrucous carcinoma (34.1%; 95% CI: 3.9–64.4) followed by well-differentiated carcinoma (26.8%; 95% CI: 19.6–34) and poorly differentiated cancer (26.7%; 95% CI: 18.7–34.7). Meanwhile, moderately differentiated cancer accounted for the lowest rate of 23.4% (16.8–30).

### 3.9. Tumor Size (T Staging) and Nodal Involvement-Based Prevalence

The rates of HPV positivity were quite similar across different tumors sizes (Table 2). For instance, the rate in T1–T2 stages was 25% compared to 25.9% in T3–T4 stages. A similar observation was noted for nodal involvement, where node-positive cancer had a rate of 16.8% compared to 17.9% for node-negative cancer. Meanwhile, the N2 stage had the highest prevalence rate of 24% while N3b had the lowest rate of 2.6%.

### 3.10. Treatment-Specific Prevalence

Significant variability in the prevalence of HPV positivity was observed for different treatment options (*p* = 0.001). Standalone chemotherapy was associated with the highest rate of HPV positivity (12.6%) followed by standalone radiotherapy (12%), surgery with radiotherapy (12%), and surgery with chemoradiation (9.2%). Meanwhile, treatment-naïve patients had the lowest rate of 3% (95% CI: 0–8.1).

### 3.11. P16-Specific Prevalence

The HPV-positive rate was higher in P16-positive patients (26.7%; 95% CI: 13.3–40%) compared to P16-negative patients (7.2%; 95% CI: 3.1–11.4%). However, this difference did not reach statistical significance (*p* = 0.151).

### 3.12. Subgroup Analyses Based on HPV-16 and HPV-18 Strains

**Figure 4:**
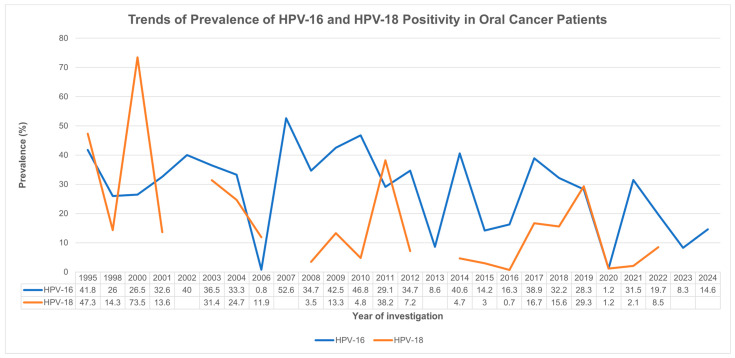
Trend analysis of HPV-16 and HPV-18 positivity in oral cavity cancer over time (1995–2024). HPV—human papillomavirus.

**Figure 5:**
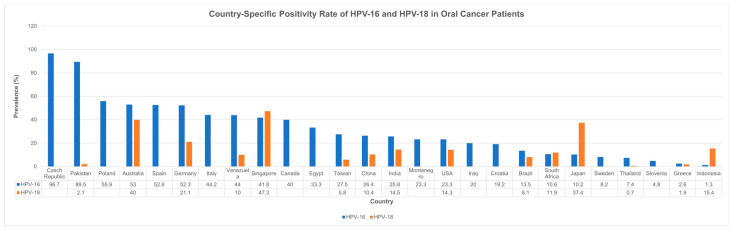
Country-specific prevalence of HPV-16 and HPV-18 positivity in oral cavity cancer. HPV—human papillomavirus; USA—United States of America.

Year- and country-specific differences in HPV prevalence between HPV-16 and HPV-18 strains are illustrated in Figure 4 and Figure 5. Although a declining trend can be observed in the prevalence of both strains over time, HPV-16 showed a mildly higher positivity rate than HPV-18 across most years. Additionally, HPV-16 showed higher positivity rates compared to the HPV-18 strain across most countries except for Japan, where HPV-18 showed predominance (37.4% vs. 10.2%).

**Figure 6:**
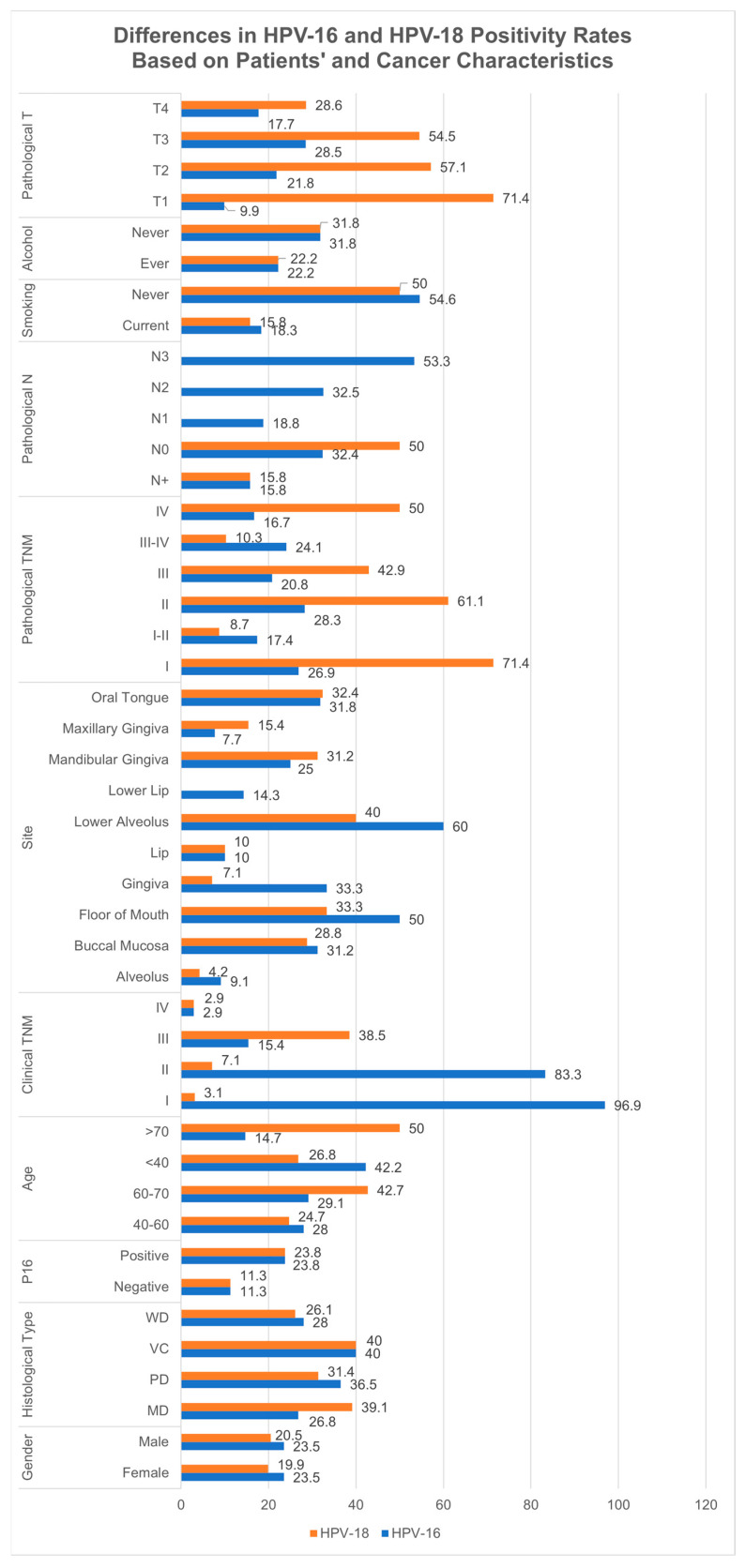
Differences in the prevalence of HPV-16 and HPV-18 positivity based on various patients’ clinicodemographic characteristics. HPV—human papillomavirus; WD—well-differentiated; VC—verrucous cancer; PD—poorly differentiated; MD—moderately differentiated.

The analysis revealed several significant differences in HPV-16 and HPV-18 positivity rates across clinicodemographic factors in oral cavity cancer patients (Tables S5–S7 and Figure 6). Among patients aged less than 40 years, HPV-16 positivity was significantly higher at 42.2% (95% CI: 22.2–62.3) compared to HPV-18 positivity at 26.8% (95% CI: 7.5–46.1). Conversely, in patients aged over 70 years, HPV-18 positivity was markedly higher at 50% (95% CI: 23.8–76.2) compared to HPV-16 positivity at 14.7% (95% CI: 0–37.4). In clinical TNM staging, HPV-16 exhibited a much higher positivity rate in stage I cancers, at 96.9% (95% CI: 88.3–100), compared to HPV-18 at 3.1% (95% CI: 0–11.7). However, in stage III cancers, HPV-18 positivity was significantly higher at 38.5% (95% CI: 12–64.9) compared to HPV-16 at 15.4% (95% CI: 0–35).

In terms of anatomical site, HPV-16 showed significantly higher positivity in cancers of the floor of the mouth at 50% (95% CI: 25.5–74.5), compared to HPV-18 at 33.3% (95% CI: 0–71.1). Conversely, in maxillary gingiva cancers, HPV-18 positivity was higher at 15.4% (95% CI: 0–35) compared to HPV-16 at 7.7% (95% CI: 0–22.2). For histological differentiation, poorly differentiated cancers exhibited slightly higher HPV-16 positivity at 36.5% (95% CI: 23.3–49.7) compared to HPV-18 at 31.4% (95% CI: 8.3–54.5). However, in moderately differentiated cancers, HPV-18 positivity was significantly higher at 39.1% (95% CI: 11.7–66.5) compared to HPV-16 at 26.8% (95% CI: 18.2–35.5). For pathological TNM staging in stage IV cancers, HPV-18 positivity was substantially higher at 50% (95% CI: 21.7–78.3) compared to HPV-16 at 16.7% (95% CI: 0–37.8). Similar positivity rates for HPV-16 and HPV-18 were observed across other patient clinicodemographic data and categories.

## 4. Discussion

### 4.1. Overview of Findings

This review synthesizes global evidence on the prevalence of HPV positivity in OCSCC and its variation across populations, time periods, detection methods, and clinicopathological strata. The pooled estimates and subgroup patterns highlight substantial heterogeneity that is partly methodological (assay and case definition) and partly epidemiological (region and case-mix). We focus the discussion on interpreting these drivers and their implications for practice and research, without revisiting the general background on OPSCC.

### 4.2. HPV Prevalence in Oral Cavity Cancer: A Global and Temporal Perspective

The prevalence of HPV positivity in oral cavity cancers demonstrated marked geographic variability, with the highest rates observed in Singapore, Venezuela, and the Czech Republic and the lowest in South Korea, Greece, and the Netherlands. These differences may be attributable to variation in risk factor exposures, healthcare access, and methodological inconsistencies across studies. The observed negative temporal trend, with declining HPV positivity rates in more recent years, might reflect improvements in tobacco and alcohol cessation programs or enhanced public health awareness about HPV vaccination, particularly in countries with robust vaccination programs [139,140,141].

This divergence from the rising HPV attribution observed in oropharyngeal cancer is likely explained, at least in part, by improved anatomic compartmentalization (reducing misclassification of tonsillar/base-of-tongue tumors as “oral cavity”) and assay standardization that together deflate earlier OCSCC estimates and yield lower, more specific recent rates. Detection methodology has shifted from heterogeneous p16-only surrogacy toward DNA/RNA-based testing and combined algorithms with more stringent positivity criteria. Simultaneously, better site assignments (distinguishing oral cavities from the oropharynx) have reduced historical misclassification. Both changes would bias calendar time trends downward for OCSCC and counsel caution against attributing the decline solely to changes in oral HPV exposure.

### 4.3. Age and Gender Differences in HPV Positivity

The prevalence of HPV showed significant age-related trends, with younger patients (<40 years) exhibiting the highest rates, which gradually declined with advancing age. This may indicate a potential role for recent changes in sexual behavior and HPV exposure patterns, particularly among younger cohorts. Although female patients exhibited slightly higher HPV positivity rates compared to males, this difference did not reach statistical significance. These findings align with prior research indicating gender parity in HPV-related oral cancers but underscore the need for targeted studies exploring potential gender-specific behavioral or biological susceptibilities [139,142,143].

### 4.4. HPV Subtype-Specific Prevalence: HPV-16 and HPV-18

This study highlights significant differences in the distribution of HPV-16 and HPV-18 positivity rates across various clinicodemographic categories. HPV-16 positivity predominated in younger patients and earlier cancer stages, whereas HPV-18 was more prevalent in older individuals and advanced disease stages. These distinctions support the hypothesis that different HPV subtypes may influence cancer pathogenesis differently, potentially due to variations in oncogenic potential and host–virus interactions. Such findings are critical for designing subtype-specific diagnostic and therapeutic strategies [140,142].

### 4.5. Cancer Site and Stage-Specific Differences

HPV positivity rates were highest in cancers of the lower alveolus and lips, with significantly lower rates in the gingivobuccal sulcus and upper gingiva. This site-specific variation may reflect differences in epithelial susceptibility to HPV infection or local microenvironmental factors influencing viral persistence. Furthermore, stage-specific analyses revealed contrasting trends across staging systems, with clinical TNM staging indicating higher positivity in early stages, while pathological and AJCC staging showed reduced prevalence in earlier stages. These discrepancies highlight the complexities of staging HPV-associated cancers and emphasize the need for standardization in reporting and classification [3,4,143].

### 4.6. Methodological and Detection Challenges

Our findings underscore the challenges in HPV detection, particularly in oral cavity cancers. While p16 immunohistochemistry is widely used as a surrogate marker, its limitations in distinguishing transcriptionally active HPV warrant caution. Incorporating more robust methods, such as E6/E7 mRNA analysis, could enhance diagnostic accuracy and reduce misclassification bias. These methodological discrepancies likely contribute to the heterogeneity observed in HPV prevalence estimates and their associations with clinical outcomes [4,140,142,144].

### 4.7. Public Health and Clinical Implications

The declining prevalence of HPV positivity, coupled with significant geographic and subtype variability, has implications for public health policies, including HPV vaccination programs. The low HPV prevalence in certain regions underscores the importance of tailoring vaccination strategies and public awareness campaigns to local epidemiological contexts. Additionally, understanding subtype-specific differences may inform personalized therapeutic approaches and improve prognostic stratification for HPV-related oral cavity cancers [140,141,143].

### 4.8. Limitations and Future Directions

Although this study synthesizes data from a large number of studies, limitations persist, including potential over- or under-reporting biases, as some countries had numerous publications on this topic while other countries barely reported any data (like Middle Eastern countries), as well as the presence of limited data from certain regions. Additionally, data on other strains of HPV were not sufficient to run meaningful analyses. Furthermore, race is a known risk factor of oral cavity and other cancer types [145], and has been associated with HPV in the literature [12]. Unfortunately, only a few studies reported data based on various races/ethnic groups [42,120]. Future research should prioritize standardization in HPV detection and reporting, alongside longitudinal studies to assess temporal trends in HPV-related oral cancers. Additionally, the integration of genomic and transcriptomic analyses could elucidate the biological mechanisms underpinning HPV-mediated carcinogenesis.

## 5. Conclusions

This study highlights significant variations in HPV prevalence across geographic regions, patient demographics, cancer sites, and stages. The findings underscore the importance of tailored prevention and treatment strategies while identifying critical gaps in current research. Future efforts should focus on harmonizing methodologies and exploring the molecular underpinnings of HPV’s role in oral cavity cancer to advance clinical care and public health interventions.

## Figures and Tables

**Table 1:** Baseline characteristics of included studies estimating the prevalence of HPV positivity in oral cancer patients (n = 122).

Author (YOP)	Country	Year of Investigation	Design	Sample Size	Cancer Location (Number)	Diagnostic Method (HPV)	Age	Gender		
							Mean	SD	Male	Female
De Abreu (2018) [54]	Brazil	2012–2015	Cross-sectional	**90**	Tongue (49), FOM (22), Other (19)	Nested PCR using MY09/MY11 and GP5+/GP6+ primers	57.9	12.73	68	22
Abreu (2020) [26]	UK	2011–2015	Prospective cohort	99	Tongue (72), FOM (9)	ISH	60.5	13.3	77	22
ADAMOPOULOU (2008) [27]	Germany	2008	Cross-sectional	102	Oral cavity cancer (68)	PCR protocol	52.1	10.3	51	51
Adilbay (2018) [28]	Kazakhistan	2015–2017	Prospective cohort	76	Oral cavity cancer (42)	PCR protocol	57.2	11.45	50	26
Afzal (2019) [29]	Pakistan	2018–2019	Cross-sectional	140	Oral cavity cancer (140)	PCR protocol	48.86	9.37	114	26
Ahmed (2019) [30]	Iraq	2019	Cross-sectional	80	Oral cavity cancer (40)	PCR protocol	-	-	24	16
Ajila (2021) [31]	India	2021	Case-control	60	Oral cavity cancer (30)	PCR protocol	58	8.86	25	5
Akhondnezhad (2018) [32]	Iran	2006–2016	Cross-sectional	83	Oral cavity cancer (83)	PCR protocol	46.2	15.5	43	40
Ali (2008) [33]	Pakistan	1991–2004	Retrospective cohort	140	Oral cavity (86), tongue (54)	PCR protocol/primers GP5/6	50	13	82	58
Alsharif (2021) [34]	Germany	2002–2011	Cross-sectional	280	Not specified	ISH	62.8	12	188	92
Vidal Loustau (2019) [136]	Switzerland	2001–2011	Retrospective cohort	155	Mobile tongue (61)	PCR protocol	66.5	13.63	107	48
Antuncov (2022) [35]	Montenegro	2012–2018	Cross-sectional	60	Tonge (22), FOM (10), lower lip (28)	PCR protocol	62	10.5	47	13
Anwar (2024) [36]	Pakistan	2017	Cross-sectional	186	Not specified	PCR protocol	-	-	-	-
Ashraf (2017) [37]	Iran	2017	Case-control	100	Oral tongue SCC (50)	nested PCR	53.54	11.19	41	59
Balaram (1995) [38]	Singapore	1995	Cross-sectional	91	Oral cavity (91)	PCR protocol	-	-	-	-
Belobrov (2017) [39]	Australia	2007–2011	Prospective cohort	46	Tongue (20), FOM (5), check mucosa (5), Mandibular Alveolus (2)	Laser capture microdissection	-	-	26	20
Bijina (2020) [41]	India	2020	Case-control	90	Oral cavity (47)	PCR protocol, gel electrophoresis	55	14.96	70	20
Boy (2006) [42]	South Africa	1998–2003	Cross-sectional	59	Oral cavity (59)	ISH/signal enhancement (Genpoint)/PCR	57.58	8.41	41	18
Božinović (2020) [43]	Serbia	2005–2006	Cross-sectional	63	Tonsil (13), Tongue (9)	ISH	54.7	4.6	39	24
Campisi (2006) [44]	Italy	2006	Cross-sectional	63	Not specified	PCR protocol	68.89	11.78	28	35
Chakrobarty (2014) [45]	India	2006–2008	Case-control	183	Oral cancer (83)	PCR protocol	50.81	10.56	136	47
Chen (2012) [46]	Taiwan	2003–2004	Cross-sectional	65	Tongue (35), buccal mucosa (20), gingiva (2), hard palate (1), FOM (7)	ISH	54.3	10.88	52	13
Chen (2016) [47]	China	2016	Cross-sectional	99	Oral cavity cancer (40)	PCR protocol	56.7	-	35	5
Chotipanich (2018) [48]	Thailand	2018	Case-control	208	Oral cavity (52)	PCR protocol	60	11.7	154	54
Chowdary (2018) [49]	India	2018	Case-control	40	Oral cavity (20)	PCR protocol	-	-	24	16
Cutilli (2016) [50]	Italy	1992–2012	Retrospective cohort	75	Not specified	PCR protocol	67	3.15	57	18
DAHLGREN (2004) [51]	Sweden	1970–2002	Cross-sectional	110	Mobile tongue (85), base of tongue (25)	PCR protocol/primers GP5/6	62.46	12.72	69	41
D’Costa (1998) [53]	India	1998	Cross-sectional	100	Buccal (57), tongue (14), FOM (2)	PCR protocol	51.3	12.2	72	28
Dhanapal (2015) [57]	India	2015	Cross-sectional	23	Buccal mucosa (8), FOM (2), tongue (1)	PCR protocol	61.5	6.5	7	7
Duncan (2013) [59]	USA	2002–2007	Cross-sectional	81	Tongue (36), FOM (11), buccal mucosa (4), lip (2)	PCR protocol/IHC	63.9	12.57	44	37
Elango (2011) [60]	India	2004–2007	Case-control	106	Oral tongue cancer (60)	PCR protocol, IHC, ISH	53.87	13.32	76	30
Emmett (2017) [62]	Australia	2006–2012	Cross-sectional	63	Tongue (48), FOM (14), Oral cavity (1)	PCR protocol	60.7	13	47	16
Emmett (2018) [61]	Australia	2018	Cross-sectional	136	Oral cavity (40)	PCR protocol	-	-	113	23
Nola-Fuchs (2012) [95]	Croatia	2012	Case-control	54	Not specified	Swab	53.9	10.1	45	9
Gan (2014) [63]	China	2009–2013	Case-control	268	Not specified	PCR protocol	-	-	-	-
Giovannelli (2006) [64]	Italy	2004	Cross-sectional	116	Oral cavity (17)	PCR protocol	58.9	12.75	49	67
Goto (2023) [66]	Japan	2009–2013	Cross-sectional	67	Tongue (34), FOM (5)	PCR protocol	-	-	54	13
Götz (2016) [67]	Germany	2009–2011	Cross-sectional	202	Not specified	IHC	57.58	10.23	145	57
Ha (2022) [69]	Maryland	1982–2000	Cross-sectional	102	Oral cavity (34)	PCR protocol	59	15.5	85	17
Harbor (2024) [70]	South Africa	2009–2019	Cross-sectional	50	Lip (50)	HybriSpot HPV Direct Flow Chip kit	61	14	38	12
Huang (2012) [72]	Taiwan	1997–2003	Cross-sectional	103	Tongue (60), lip (1), mouth floor (6)	PCR protocol	94.4	10.9	96	7
Huang (2017) [71]	Taiwan	2017	Cross-sectional	85	Not specified	PCR protocol	-	-	78	7
Ibieta (2005) [8]	Mexico	1999–2001	Cross-sectional	50	Tongue (13), mouth of floor (4)	PCR protocol	-	-	36	14
Ishibashi (2011) [73]	Japan	2011	Cross-sectional	107	Oral cavity (50)	PCR protocol/using consensus primers (My09/My11, GP5?/GP6?)	59.2	13.72	57	50
Jaber (2019) [74]	Saudi Arabia	2010–2014	Retrospective cohort	45	Not specified	ISH	60.25	-	24	21
JALOULI (2010) [75]	India	2010	Cross-sectional	74	Tongue (18), buccal (12), lip (6)	PCR protocol	55.3	10.7	59	15
Jalouli (2012) [76]	Sweden	2012	Cross-sectional	155	Tongue (41), FOM (23)	PCR protocol	63.3	-	-	-
JitAni (2015) [77]	India	2010–2013	Cross-sectional	31	Not specified	PCR protocol/ISH	-	-	16	15
Kaminagakura (2012) [78]	Brazil	1970 to 2006	Case-control	114	Tongue (23), buccal (1)	PCR protocol/IHC	34	5.4	83	33
KANSKY (2003) [9]	Slovenia	1994–1998	Case-control	124	Oral cavity (62)	PCR protocol	58.2	7.3	55	7
Grewal (2018) [68]	India	2011–2014	Cross-sectional	47	Tongue (23), lip (4), buccal (9)	nested PCR	-	-	36	11
Khanna (2009) [79]	India	2007–2009	Case-control	120	Not specified	PCR protocol	50.6	-	90	30
Khovidhunkit (2008) [80]	Thailand	2008	Cross-sectional	65	Buccal mucosa (11)	PCR protocol	58.22	13.06	15	50
Kim (2018) [81]	South Korea	2010–2015	Retrospective cohort	187	Tongue (54), gum (80)	DNA chip kit	64	11.9	116	71
Klozar (2008) [82]	Czech Republic	2001–2005	Cross-sectional	81	Tonsil (51), oral (10), tongue (4), base of tongue (10)	PCR protocol	-	-	51	30
Komolmala (2020) [10]	Thailand	1999–2019	Cross-sectional	403	Tongue (46), FOM (8)	PCR protocol	66	-	78	94
Kouketsu (2015) [83]	Japan	2012–2013	Cross-sectional	174	Tongue (90), gingiva (43), buccal (22), FOM (7), lip (11)	PCR protocol	67.6	12.7	76	98
Kulkarni (2011) [84]	India	2009–2010	Cross-sectional	490	Oral cavity (34)	PCR protocol	-	-	-	-
Bhawal (2007) [40]	Japan	2007	Cross-sectional	22	Oral cavity (22)	PCR protocol/PT-PCR	66.6	12.6	13	9
Lee (2012) [85]	Taiwan	2004–2006	Prospective cohort	333	Not specified	PCR protocol	-	-	316	17
Lee (2015) [86]	Taiwan	2004–2011	Retrospective cohort	1002	Tongue (322), lip (35), FOM (31)	PCR protocol	-	-	938	64
Liang (2008) [87]	China	2004–2006	Cross-sectional	51	Oral tongue (51)	PCR protocol	59.5	12.4	31	20
Lukesova (2014) [88]	Czech Republic	2014	Cross-sectional	60	Oral cavity (5)	PCR protocol	56.5	-	54	6
Machado (2010) [11]	Canada	1995–2007	Retrospective cohort	92	Oral cavity, tongue, FOM, palate, buccal mucosa and gingiva (53)	PCR protocol	-	-	64	28
Makvandi (2022) [15]	Iran	2013–2019	Cross-sectional	166	Oral tongue (140), base of tongue (22), tonsils (4)	Nested PCR	53.23	15.9	144	22
Matzow (2009) [89]	Sweden	2009	Cross-sectional	54	Tongue (11), FOM (7), gingiva (10), buccal (2)	PCR protocol	-	-	-	-
De Menezes (2022) [55]	Brazil	2019	Cross-sectional	101	Tongue (19), lip (16), gingiva (46)	PCR/”Inno-Lipa Genotyping Extra II System	-	-	46	55
Montaldo (2010) [90]	Italy	2007–2008	Case-control	120	Not specified	PCR protocol	61.7	13.3	72	48
More (2020) [91]	Saudi Arabia	2020	Cross-sectional	45	Oral cavity (30)	PCR protocol	-	-	31	14
NAGPAL (2001) [92]	India	2001	Case-control	110	Tongue (6), lip (4)	PCR assay	-	-	68	42
Naqvi (2020) [93]	Pakistan	2015–2017	Cross-sectional	58	Tongue (17), lip (11), buccal mucosa (24)	PCR protocol	42	12	48	10
Nauta (2021) [94]	The Netherlands	2008–2014	Retrospective cohort	940	Tongue (451), FOM (268)	PCR protocol	64.86	12	551	389
Nekić (2022) [16]	Croatia	2022	Retrospective cohort	99	Oral cavity (26)	PCR protocol	-	-	89	10
OLIVEIRA (2003) [96]	Brazil	2008	Retrospective cohort	87	Tongue (22), lip (13)	PCR protocol	-	-	73	14
Ostwald (2003) [97]	Germany	2003	Cross-sectional	267	Intraorally (93), lips (21)	PCR protocol	58.57	-	186	81
PALMIER (2011) [98]	Italy	1990–2007	Case-control	278	Oral cavity	RT-PCR	-	-	-	-
Panneerselvam (2019) [99]	India	2019	Cross-sectional	30	Not specified	PCR protocol	46.7	-	27	3
Panzarella (2021) [100]	Italy	2021	Cross-sectional	40	Not specified	PCR protocol	66.5	14.1	17	23
Parshad (2015) [101]	India	2015	Prospective cohort	50	Tonsil (15), base of tongue (16)	PCR protocol	55.32	10.2	44	6
Patel (2015) [102]	India	2015	Cross-sectional	149	Tongue (21), buccal (39)	PCR protocol	48.3	10.8	84	65
Premoli-De-Percoco (2001) [108]	Venezuela	2001	Cross-sectional	50	Tongue (18), buccal mucosa (7), FOM (7)	PCR protocol	56.3	-	0	50
Petito (2017) [103]	Brazil	2005–2007	Cross-sectional	82	Oral cavity (39)	PCR protocol	-	-	64	18
Petrovic (2023) [13]	Serbia	2018–2022	Cross-sectional	90	Tongue (19), lip (4), buccal (4)	PCR protocol	62.95	-	48	42
Phusingha (2016) [104]	Thailand	2005–2010	Case-control	191	Tongue (20), lip (16), FOM (16)	Reverse line blot hybridization (RLBH)	-	-	115	76
POLZ (2010) [106]	Poland	1998–2004	Cross-sectional	60	Oral cavity (21)	PCR protocol	57.5	-	54	6
Polz-Gruszka (2015) [107]	Poland	2006–2009	Retrospective cohort	154	Oral cavity (92)	PCR protocol	56.8	8.8	131	23
Pongsapich (2016) [17]	Thailand	2010–2012	Cross-sectional	46	Not specified	PCR protocol	59.6	15.16	29	17
Ravi Prakash (2024) [111]	India	2020–2022	Retrospective cohort	100	Not specified	ISH or PCR.	58.75	8.1	74	26
Purwanto (2019) [109]	Indonesia	2003–2013	Cross-sectional	78	Tongue (58), lip (6), buccal (2)	PCR protocol	47.08	14.15	47	31
Rahbarnia (2019) [110]	Iran	2012–2014	Case-control	60	Tongue (30)	PCR protocol	61.3	13.7	26	34
González-Ramírez (2013) [65]	Mexico	2007–2011	Case-control	400	Tongue (47), palate (11), buccal (1), Gingival (21)	PCR protocol	-	-	-	-
Delgado Ramos (2018) [56]	Ecuador	2006–2011	Cross-sectional	53	Tongue (100%)	PCR protocol	61.8	17.3	29	24
Rivero (2006) [112]	Brazil	2006	Cross-sectional	40	Lip (20), Tongue (14), gingiva (3), FOM (2) and palate (1)	PCR protocol	57	13.6	32	8
Rodríguez-Santamarta (2016) [113]	Spain	1996–2007	Retrospective cohort	125	Tongue (51), FOM (37), buccal (7)	PCR protocol/ ISH	58.6	14.4	82	43
ROMANITAN (2008) [14]	Greece	1986–2007	Cross-sectional	115	Tonsil (31), tongue (38)	PCR protocol	62	7.9	-	-
Rout (2024) [114]	India	2024	Cross-sectional	140	Not specified	PCR protocol	54.5	-	117	23
Rungraungrayabkul (2022) [115]	Thailand	2013–2019	Retrospective cohort	81	Tongue (24) buccal mucosa (11) lip (5)	PCR protocol	-	-	32	49
Saini (2010) [116]	Malaysia	2010	Case-control	210	Tongue (29), lip (1)	GP5+/GP6+ in a nested PCR	49.12	13.4	109	101
Schwartz (2001) [117]	USA	1988–1995	Cross-sectional	254	Tongue (81), tonsil (44)	PCR protocol	54.2	-	163	91
Shima (2000) [118]	Japan	1991–1996	Cross-sectional	46	Tongue (27), buccal (3), FOM (3)	PCR protocol	50	14	32	14
Sichero (2024) [119]	brazil	2015–2019	Cross-sectional	146	Oral cavity (89)	PCR protocol	-	-	118	28
Simonato (2008) [120]	Brazil	1991–2005	Cross-sectional	29	Not specified	PCR protocol/GP5+⁄GP6+ (35)	-	-	27	2
Singh (2015) [122]	India	2013–2015	Prospective cohort	250	Buccal mucosa (127), FOM (4)	Real-Time PCR, Conventional PCR/IHC	-	-	200	50
Singh (2016) [121]	India	2013–2014	Prospective cohort	43	Not specified	PCR protocol	45.56	10.04	37	6
Smith (1998) [123]	USA	1994–1996	Case-control	298	Not specified	PCR protocol	-	-	198	100
Soares (2007) [124]	Brazil	2000–2003	Cross-sectional	75	Tongue (20), FOM (17), lips (14)	PCR protocol	65.45	13.2	49	26
Sri (2021) [125]	India	2010–2012	Cross-sectional	40	Not specified	Qiagen QIAamp DNA tissue Kit (Qiagen Inc., USA).	-	-	-	-
Dirasantchu (2015) [58]	India	2015	Case-control	35	Buccal mucosa (10), tongue (5), alveolus (4), retromolar (3), buccal sulcus (1)	PCR protocol	-	-	24	11
Taberna (2017) [126]	USA	July 1905	Prospective cohort	262	Oral cavity (90)	ISH	-	-	213	49
Tachezy (2005) [127]	Czech Republic	2000–2003	Cross-sectional	68	Tongue (5), tonsil (8)	PCR protocol	57	-	54	14
Tang (2020) [128]	The Netherlands	2020	Cross-sectional	183	Not specified	nested PCR	-	-	118	65
Tangthongkum (2024) [129]	Thailand	2012–2021	Retrospective cohort	381	Not specified	PCR protocol	-	-	232	149
Tealab (2009) [130]	Egypt	2008–2015	Retrospective cohort	99	tongue (48), lip (45)	PCR protocol/ISH	57.2	13	55	44
Tokuzen (2021) [131]	Japan	2004–2013	Cross-sectional	100	Tongue (36), mandibular gingiva (31), maxillary gingiva (13), FOM (9), buccal mucosa (9), or lower lip (2)	RT-qPCR	68.2	10.08	54	46
Dalla Torre (2018) [52]	Australia	2008–2012	Retrospective cohort	106	Not specified	PCR protocol	58.9	7.9	71	35
TSIMPLAKI (2014) [132]	Greece	2012–2013	Cross-sectional	53	Not specified	PCR protocol	51	12.4	39	14
Valls-Ontanón (2007) [133]	Spain	2010–2011	Retrospective cohort	155	Tongue (47), buccal (11), lip (8)	PCR protocol	72.7	13.4	107	48
Vanshika (2021) [134]	India	2018–2019	Cross-sectional	216	Not specified	(RT-PCR)	45.6	-	172	44
Pintos Vega (2002) [105]	Canada	1997–2001	Case-control	201	Tongue except base (21), FOM (12), lips (1)	PCR protocol/DNA sequencing	62.7	-	143	58
Verma (2018) [135]	India	2018	Case-control	100	Tongue (16), buccal (12). lip (3)	PCR	47.69	6.73	-	-
Yang (2019) [137]	China	2016–2017	Case-control	163	Tongue (70), buccal (40), FOM (3)	IHC	81.5	12	76	87
Zhang (2004) [138]	China	1997–1999	Case-control	113	Tongue (35), buccal (14), FOM (10)	PCR protocol	-	-	72	41

YOP—year of publication; YOI—year of investigation; SD—standard deviation; USA—United States of America; UK—United Kingdom; PCR—polymerase chain reaction; IHC—Immunohistochemical Staining; ISH—in situ hybridization; RT-PCR—real-time PCR.

**Table 2:** The prevalence of HPV positivity in oral cavity cancer stratified by patients’ clinical/cancer characteristics and management type.

Group	Prevalence (%)	95% CI	Studies	Q	*p*-Value	Tau^2^	I^2^ (%)	H2
Gender								
Female	24.6	19.3–29.8	65	1022.39	0.000	0.038	96.39	27.71
Male	23.5	18.8–28.2	66	1201.37	0.000	0.035	96.84	31.63
Age								
<40	29.7	20.3–39	20	75.32	0.000	0.026	81.45	5.39
40–60	25.4	19.8–30.9	61	812.97	0.000	0.043	95.15	20.61
60–70	24.3	17.4–31.3	26	242.16	0.000	0.027	91.43	11.67
>70	23.8	8.6–39.1	8	42.02	0.000	0.037	90.78	10.85
Smoking								
Current	27.2	18.4–36	31	584.64	0.000	0.058	97.58	41.28
Ever	23.3	4.1–42.4	7	126.02	0.000	0.064	97.5	39.95
Former	9.4	0–19	4	1.87	0.599	0.000	0	1
Never	25.4	18.1–32.8	33	297.04	0.000	0.039	93.98	16.62
Alcohol								
Ever	22.7	14.9–30.4	24	207.95	0.000	0.032	94.81	19.26
Excessive	12.2	7.2–17.2	1	0.00		0.000		
Never	21.8	13.9–29.8	26	254.81	0.000	0.036	95.68	23.15
Histological Type								
MD	23.4	16.8–30	38	542.57	0.000	0.037	96.61	29.48
PD	26.7	18.7–34.7	37	257.32	0.000	0.039	88.89	9
VC	34.1	3.9–64.4	4	22.09	0.000	0.080	90.88	10.97
WD	26.8	19.6–34	36	659.14	0.000	0.043	97.12	34.75
AJCC								
I	7.8	0–15.8	2	0.17	0.676	0.000	0	1
II	3.3	0–8.2	3	0.95	0.622	0.000	0	1
III	3.9	0–9.2	3	0.47	0.791	0.000	0.01	1
IV	10.3	5.5–15.1	3	0.31	0.859	0.000	0.01	1
Site								
Lip	25	14.7–35.3	19	79.63	0.000	0.034	78.07	4.56
Lower Lip	14.8	6–23.6	5	1.43	0.838	0.000	0	1
Upper Lip	16.7	0–58.8	1	0.00		0.000		
Gingiva	18.2	10.9–25.5	20	64.75	0.000	0.018	83.7	6.14
Lower Gingiva	18.8	2.3–35.3	2	2.1	0.147	0.007	52.49	2.1
Upper Gingiva	3.9	0–10.5	3	0.81	0.665	0.000	0	1
Mandibular Gingiva	24.6	3.3–45.9	5	12.93	0.012	0.035	67.33	3.06
Maxillary Gingiva	23.1	8.9–37.2	4	4.05	0.256	0.000	0	1
Alveolus	24.5	0–56.1	4	26.89	0.000	0.094	91.53	11.81
Lower Alveolus	29.5	0–76.5	3	32.68	0.000	0.166	97.98	49.6
Upper Alveolus	7.1	0–26.2	1	0.00		0.000		
Oral Tongue	22.7	16.7–28.7	51	504.77	0.000	0.042	95.38	21.67
Mobile Tongue	11.2	0–24.1	5	21.66	0.000	0.018	97.43	38.92
Tongue Border	17.1	0–34.9	2	0.16	0.687	0.000	0	1
Buccal Mucosa	20.9	14.2–27.6	39	423.85	0.000	0.036	93.46	15.3
Floor of Mouth	14.8	10.7–19	38	91.97	0.000	0.007	66.44	2.98
Gingivobuccal sulcus	4.7	0–13	2	0.45	0.504	0.000	0	1
Hard Palate	18.9	10.8–26.9	24	54.4	0.000	0.019	61.98	2.63
Retromolar Trigone	10.5	5–16	17	31.82	0.011	0.004	45.64	1.84
Vestibulum of Mouth	0.4	0–1.5	1	0.00		0.000		
Waldeyer ring	25.8	10.4–41.2	1	0.00		0.000		
Pathological TNM								
I	31.9	19.7–44.2	18	254.33	0.000	0.060	95.98	24.9
II	36.4	24.3–48.6	18	194.61	0.000	0.059	92.81	13.92
III	32.3	20.1–44.5	17	156.03	0.000	0.056	90.88	10.97
IV	29.1	17.3–40.8	15	129.22	0.000	0.045	93.4	15.15
I–II	28.8	19.1–38.4	25	556.59	0.000	0.055	97.47	39.6
III–IV	27.7	19.3–36.1	23	282.89	0.000	0.038	95.14	20.59
Pathological T								
T1	25.4	13.5–37.2	18	224.66	0.000	0.056	97.82	45.9
T2	25.9	15–36.8	19	415.85	0.000	0.053	98.56	69.44
T3	28.4	16.5–40.3	20	241.49	0.000	0.066	96.11	25.71
T4	25.9	14.9–37	19	312.68	0.000	0.052	96.05	25.31
T1–T2	25	15.8–34.1	25	621.71	0.000	0.050	98.74	79.1
T3–T4	25.9	16.8–35.1	25	582.91	0.000	0.051	97.54	40.6
Pathological N								
N0	17.9	9.5–26.4	18	291.31	0.000	0.029	97.03	33.68
N+	16.8	10.8–22.8	18	153.19	0.000	0.013	90.42	10.44
N1	18.3	5.5–31.1	10	86.29	0.000	0.036	93.42	15.2
N2	24	15.4–32.5	9	14.09	0.079	0.007	48.27	1.93
N2a	11.7	0–40.6	2	2.67	0.102	0.031	62.52	2.67
N2b	4.4	0–9.5	2	1.8	0.180	0.001	44.33	1.8
N2c	9	2.6–15.4	2	0.18	0.672	0.000	0	1
N3	28.7	11.1–46.3	6	8.46	0.132	0.018	38.27	1.62
N3a	16.7	0–58.8	1	0.00		0.000		
N3b	2.6	0–5.7	2	0.39	0.534	0.000	0	1
N4	16.7	0–46.5	1	0.00		0.000		
Clinical TNM								
I	41.8	3.3–80.4	4	193.14	0.000	0.146	97.55	40.81
I–II	24	5.6–42.5	10	328.29	0.000	0.085	97.48	39.61
II	27.7	3.7–51.8	5	29.47	0.000	0.067	94.05	16.82
III	12.4	1.7–23.1	4	6.94	0.074	0.006	56.97	2.32
III–IV	12.7	8.5–16.8	10	20.66	0.014	0.002	58.14	2.39
IV	10.4	1.7–19.2	4	9.75	0.021	0.006	74.24	3.88
Clinical N								
N0	12	3.4–20.5	8	144.06	0.000	0.013	97.72	43.94
N+	16.2	2.4–29.9	8	158.24	0.000	0.038	98.24	56.93
N1	8	1.4–14.7	6	22.91	0.000	0.005	80.84	5.22
N2	9.8	2.9–16.7	6	50.75	0.000	0.006	88.6	8.77
N3	7	0–23.4	3	1.73	0.422	0.007	13.79	1.16
Management								
Chemoradiation	10.5	6–15	5	2.15	0.709	0.000	0.02	1
Chemotherapy	12.6	6.2–19	2	0.00	0.980	0.000	0	1
Radiotherapy	12	1.2–22.9	4	16.22	0.001	0.007	75.15	4.02
Surgery alone	7.1	2.1–12.1	6	52.12	0.000	0.003	93.99	16.63
Surgery plus chemoradiation	9.2	2.4–16	5	19.63	0.001	0.004	79.2	4.81
Surgery plus radiotherapy	12	4.1–19.9	5	24.7	0.000	0.006	86.21	7.25
Treatment-naïve	3	0–8.1	2	0.34	0.559	0.000	0	1
P16 Positivity								
Negative	7.2	3.1–11.4	13	55.19	0.000	0.004	82.66	5.77
Positive	26.7	13.3–40	13	154.28	0.000	0.050	95.15	20.6

MD—moderately differentiated; WD—well-differentiated; PD—poorly differentiated.

## Data Availability

The analyzed dataset was derived from data published in the literature; however, the full dataset can be shared by the corresponding author upon reasonable request.

## Supplementary Materials

The following supporting information can be downloaded at https://www.mdpi.com/article/10.3390/cancers17172870/s1. Table S1. The detailed search criteria employed in the literature search [date of search: 9 October 2024]; Table S2. A summary of the methodological quality of included studies using the National Institute of Health quality assessment tool; Table S3. The prevalence of total HPV positivity in oral cancer patients stratified by the year of investigation; Table S4. The prevalence of total HPV positivity in oral cancer patients stratified by the country of investigation; Table S5. Year-specific differences in HPV-16 and HPV-18 positivity in oral cavity cancer patients; Table S6. Country-specific differences in positivity rates of HPV-16 and HPV-18 in oral cavity cancer patients; Table S7. Differences in HPV-16 and HPV-18 positivity rates across various patient groups.

## Abbreviations

The following abbreviations are used in this manuscript:

AJCC	American Joint Committee on Cancer
CI	Confidence interval
DNA	Deoxyribonucleic acid
E6/E7	Early genes 6 and 7 of HPV
FISH	Fluorescence in Situ Hybridization
HPV	Human papillomavirus
IHC	Immunohistochemistry
I^2^	I-squared
LD	Linear dichroism
MD	Moderately differentiated
OPC	Oropharyngeal cancer
OPSCC	Oropharyngeal squamous cell carcinoma
OR	Odds ratio
OS	Overall survival
P16	Cyclin-dependent kinase inhibitor 2A
PD	Poorly differentiated
PECO	Population, exposure, comparison, and outcomes
PRISMA	Preferred Reporting Items for Systematic Reviews and Meta-Analyses
REML	Restricted maximum likelihood
RNA	Ribonucleic acid
ROC	Receiver operating characteristics
SCC	Squamous cell carcinoma
TNM	Tumor, node, and metastasis
VC	Verrucous carcinoma
WD	Well differentiated

## References

[1] Marur S., D’Souza G., Westra W.H., Forastiere A.A. (2010). HPV-associated head and neck cancer: A virus-related cancer epidemic. Lancet Oncol..

[2] Lechner M., Liu J., Masterson L., Fenton T.R. (2022). HPV-associated oropharyngeal cancer: Epidemiology, molecular biology and clinical management. Nat. Rev. Clin. Oncol..

[3] Christensen J.T., Grønhøj C., Zamani M., Brask J., Kjær E.K.R., Lajer H., von Buchwald C. (2019). Association between oropharyngeal cancers with known HPV and p16 status and cervical intraepithelial neoplasia: A Danish population-based study. Acta Oncol..

[4] Isayeva T., Li Y., Maswahu D., Brandwein-Gensler M. (2012). Human papillomavirus in non-oropharyngeal head and neck cancers: A systematic literature review. Head Neck Pathol..

[5] Zokirovna O.A. (2023). The incidence of cancer of the oral cavity and pharynx in The Bukhara Region. Int. J. Integr. Mod. Med..

[6] Lu Y., Sobue T., Kitamura T., Matsuse R., Kitamura Y., Matsuo K., Ito H., Oze I., Shimazu T., Yamaji T. (2018). Cigarette smoking, alcohol drinking, and oral cavity and pharyngeal cancer in the Japanese: A population-based cohort study in Japan. Eur. J. Cancer Prev..

[7] Lai K., Killingsworth M., Matthews S., Caixeiro N., Evangelista C., Wu X., Wykes J., Samakeh A., Forstner D., Niles N. (2017). Differences in survival outcome between oropharyngeal and oral cavity squamous cell carcinoma in relation to HPV status. J. Oral Pathol. Med..

[8] Ibieta B.R., Lizano M., Fras-Mendivil M., Barrera J.L., Carrillo A., Ma Ruz-Godoy L., Mohar A. (2005). Human papilloma virus in oral squamous cell carcinoma in a Mexican population. Oral Surg. Oral Med. Oral Pathol. Oral Radiol. Endod..

[9] Kansky A.A., Poljak M., Seme K., Kocjan B.J., Gale N., Luzar B., Golouh R. (2003). Human papillomavirus DNA in oral squamous cell carcinomas and normal oral mucosa. Acta Virol..

[10] Komolmalai N., Pongsiriwet S., Lertprasertsuke N., Lekwanavijit S., Kintarak S., Phattarataratip E., Subarnbhesaj A., Dhanuthai K., Chaisuparat R., Iamaroon A. (2020). Human Papillomavirus 16 and 18 Infection in Oral Cancer in Thailand: A Multicenter Study. Asian Pac. J. Cancer Prev..

[11] Machado J., Reis P.P., Zhang T., Simpson C., Xu W., Perez-Ordonez B., Goldstein D.P., Brown D.H., Gilbert R.W., Gullane P.J. (2010). Low prevalence of human papillomavirus in oral cavity carcinomas. Head Neck Oncol..

[12] Osazuwa-Peters N., Adjei Boakye E., Rohde R.L., Ganesh R.N., Moiyadi A.S., Hussaini A.S., Varvares M.A. (2019). Understanding of risk factors for the human papillomavirus (HPV) infection based on gender and race. Sci. Rep..

[13] Petrović A., Čanković M., Avramov M., Popović Ž.D., Janković S., Mojsilović S. (2023). High-Risk Human Papillomavirus in Patients with Oral Carcinoma and Oral Potentially Malignant Disorders in Serbia-A Pilot Study. Medicina.

[14] Romanitan M., Näsman A., Ramqvist T., Dahlstrand H., Polykretis L., Vogiatzis P., Vamvakas P., Tasopoulos G., Valavanis C., Arapantoni-Dadioti P. (2008). Human papillomavirus frequency in oral and oropharyngeal cancer in Greece. Anticancer Res..

[15] Makvandi M., Jalilian S., Faghihloo E., Khanizadeh S., Ramezani A., Bagheri S., Mirzaei H. (2022). Prevalence of Human Papillomavirus and Co-Infection with Epstein-Barr Virus in Oral and Oropharyngeal Squamous Cell Carcinomas. Asian Pac. J. Cancer Prev..

[16] Nekić I., Medić A., Puntarić D., Nemeth Blažić T., Šikić N.L., Konjevoda S., Nonković D., Sambunjak J., Dželalija B. (2022). Prevalence of Human Papillomavirus in Laryngeal, Oropharyngeal and Oral Cavity Carcinomas in Zadar County, Croatia. Infektološki Glas..

[17] Pongsapich W., Jotikaprasardhna P., Lianbanchong C., Phumchan A., Siritantikorn S., Chongkolwatana C. (2016). Human Papillomavirus Infection in Oral Cavity and Oropharyngeal Cancers: Are They the Same Story?. J. Med. Assoc. Thail. Chotmaihet Thangphaet.

[18] Page M.J., McKenzie J.E., Bossuyt P.M., Boutron I., Hoffmann T.C., Mulrow C.D., Shamseer L., Tetzlaff J.M., Akl E.A., Brennan S.E. (2021). The PRISMA 2020 statement: An updated guideline for reporting systematic reviews. BMJ.

[19] Shea B.J., Hamel C., Wells G.A., Bouter L.M., Kristjansson E., Grimshaw J., Henry D.A., Boers M. (2009). AMSTAR is a reliable and valid measurement tool to assess the methodological quality of systematic reviews. J. Clin. Epidemiol..

[20] Muka T., Glisic M., Milic J., Verhoog S., Bohlius J., Bramer W., Chowdhury R., Franco O.H. (2020). A 24-step guide on how to design, conduct, and successfully publish a systematic review and meta-analysis in medical research. Eur. J. Epidemiol..

[21] Abdelaal A., Eltaras M.M., Katamesh B.E., Serhan H.A., Farahat R.A., Badr H., Abdelazeem B. (2023). The prevalence and presentation patterns of microcystic macular oedema: A systematic review and meta-analysis of 2128 glaucomatous eyes. Eye.

[22] Amir-Behghadami M., Janati A. (2020). Population, Intervention, Comparison, Outcomes and Study (PICOS) design as a framework to formulate eligibility criteria in systematic reviews. Emerg. Med. J..

[23] Mavridis D., Salanti G., Furukawa T.A., Cipriani A., Chaimani A., White I.R. (2019). Allowing for uncertainty due to missing and LOCF imputed outcomes in meta-analysis. Stat. Med..

[24] Sedgwick P. (2013). Meta-analyses: Heterogeneity and subgroup analysis. BMJ.

[25] Lin L., Chu H. (2018). Quantifying publication bias in meta-analysis. Biometrics.

[26] Abreu P.M., Valle I.B., Damasceno T.C.D., Có A.C.G., Pansini P.F., Podestá J.R.V., Souza E.D., Rocha R.M., Curado M.P., Mehanna H. (2020). Human Papillomavirus E6/E7 mRNA detection by in situ hybridization in oral cavity squamous cell carcinoma. Arch. Oral Biol..

[27] Adamopoulou M., Vairaktaris E., Panis V., Nkenke E., Neukam F.W., Yapijakis C. (2008). HPV detection rate in saliva may depend on the immune system efficiency. In Vivo.

[28] Adilbay D., Adilbayev G., Kidirbayeva G., Shipilova V., Sadyk Z., Koyanbekova G., Sokolenko E., Klozar J. (2018). HPV infection and P16 expression in oral and oropharyngeal cancer in Kazakhstan. Infect. Agents Cancer.

[29] Afzal A., Liaqat R., Shafqat F., Kalsoom F., Loya A. (2019). The Frequency of Human Papilloma Virus Related Oral Squamous Cell Carcinomas by P16 Immuno Histochemical Stain. Med. Forum Mon..

[30] Ahmed S.M., Jabar S.K. (2019). Prevalence of human papillomavirus in oral and laryngeal squamous cell carcinoma: A comparative study by polymerase chain reaction. Med. Sci..

[31] Ajila V., Babu S., Shetty V., Shetty P., Devegowda D., Ramesh P., Natarajan S. (2021). Human papillomavirus in oral squamous cell carcinoma: An institutional study. Clin. Cancer Investig. J..

[32] Akhondnezhad M., Haghshenas M.R., Ghasemi M., Mousavi T. (2018). The prevalence and genotyping of human papillomavirus in patients with oral tumors in health centers and clinics of Mazandaran in Iran. Virusdisease.

[33] Ali S., Awan M., Ghaffar S., Salahuddin I., Khan S., Mehraj V., Pervez S. (2008). Human papillomavirus infection in oral squamous cell carcinomas: Correlation with histologic variables and survival outcome in a high risk population. Oral Surg..

[34] Alsharif U., Hofmann M., Gebhard M., Tharun L., Rades D., Sieg P., Hakim S.G. (2021). Double Positivity for HPV DNA/P16(INK4a) Does Not Influence Survival of Patients with Oral Squamous Cell Carcinoma. Anticancer Res..

[35] Antunović M., Lopičić M., Vučković L., Raonić J., Mugoša S. (2022). Prevalence and clinical implications of the HPV16 infection in oral cancer in Montenegro—Evidence to support the immunization program. Acta Microbiol. Immunol. Hung..

[36] Anwar N., Chundriger Q., Awan S., Moatter T., Ali T.S., Abdul Rasheed M., Pervez S. (2024). Prevalence of high-risk human papillomavirus in oral squamous cell carcinoma with or without chewing habits. PLoS ONE.

[37] Ashraf M.J., Hosseini S., Monabati A., Valibeigi B., Khademi B., Abedi E., Azarpira N. (2017). The Prevalence of Human Papilloma Virus in Squamous Cell Carcinoma of Oral Tongue. Iran. J. Pathol..

[38] Balaram P., Nalinakumari K.R., Abraham E., Balan A., Hareendran N.K., Bernard H.U., Chan S.Y. (1995). Human papillomaviruses in 91 oral cancers from Indian betel quid chewers--high prevalence and multiplicity of infections. Int. J. Cancer.

[39] Belobrov S., Cornall A.M., Young R.J., Koo K., Angel C., Wiesenfeld D., Rischin D., Garland S.M., McCullough M. (2018). The role of human papillomavirus in p16-positive oral cancers. J. Oral Pathol. Med..

[40] Bhawal U.K., Sugiyama M., Nomura Y., Sawajiri M., Tsukinoki K., Ikeda M.A., Kuniyasu H. (2007). High-risk human papillomavirus type 16 E7 oncogene associates with Cdc25A over-expression in oral squamous cell carcinoma. Virchows Arch. Int. J. Pathol..

[41] Bijina B.R., Ahmed J., Shenoy N., Ongole R., Shenoy S., Baliga S. (2016). Detection of human papilloma virus in potentially malignant and malignant lesions of the oral cavity and a study of associated risk factors. South Asian J. Cancer.

[42] Boy S., Van Rensburg E.J., Engelbrecht S., Dreyer L., van Heerden M., van Heerden W. (2006). HPV detection in primary intra-oral squamous cell carcinomas--commensal, aetiological agent or contamination?. J. Oral Pathol. Med..

[43] Božinović K., Sabol I., Rakušić Z., Jakovčević A., Šekerija M., Lukinović J., Prgomet D., Grce M. (2019). HPV-driven oropharyngeal squamous cell cancer in Croatia—Demography and survival. PLoS ONE.

[44] Campisi G., Giovannelli L., Calvino F., Matranga D., Colella G., Di Liberto C., Capra G., Leao J.C., Lo Muzio L., Capogreco M. (2006). HPV infection in relation to OSCC histological grading and TNM stage. Evaluation by traditional statistics and fuzzy logic model. Oral Oncol..

[45] Chakrobarty B., Roy J.G., Majumdar S., Uppala D. (2014). Relationship among tobacco habits, human papilloma virus (HPV) infection, p53 polymorphism/mutation and the risk of oral squamous cell carcinoma. J. Oral Maxillofac. Pathol..

[46] Chen S.F., Yu F.S., Chang Y.C., Fu E., Nieh S., Lin Y.S. (2012). Role of human papillomavirus infection in carcinogenesis of oral squamous cell carcinoma with evidences of prognostic association. J. Oral Pathol. Med..

[47] Chen X.J., Sun K., Jiang W.W. (2016). Absence of high-risk HPV 16 and 18 in Chinese patients with oral squamous cell carcinoma and oral potentially malignant disorders. Virol. J..

[48] Chotipanich A., Siriarechakul S., Mungkung O.O. (2018). Role of high-risk human papillomavirus in the etiology of oral and oropharyngeal cancers in Thailand: A case-control study. SAGE Open Med..

[49] Chowdary S.D., Sekhar P.C., Kattapagari K.K., Mani Deepthi C.H., Neelima D., Reddy B.V.R. (2018). A study to assess expression of human papillomavirus types 16 and 18 in oral squamous cell carcinoma using polymerase chain reaction. J. Oral Maxillofac. Pathol..

[50] Cutilli T., Leocata P., Dolo V., Altobelli E. (2016). Association between p53 status, human papillomavirus infection, and overall survival in advanced oral cancer after resection and combination systemic treatment. Br. J. Oral Maxillofac. Surg..

[51] Dahlgren L., Dahlstrand H.M., Lindquist D., Högmo A., Björnestål L., Lindholm J., Lundberg B., Dalianis T., Munck-Wikland E. (2004). Human papillomavirus is more common in base of tongue than in mobile tongue cancer and is a favorable prognostic factor in base of tongue cancer patients. Int. J. Cancer.

[52] Dalla Torre D., Burtscher D., Soelder E., Offermanns V., Rasse M., Puelacher W. (2018). Human papillomavirus prevalence in a Mid-European oral squamous cell cancer population: A cohort study. Oral Dis..

[53] D’Costa J., Saranath D., Dedhia P., Sanghvi V., Mehta A.R. (1998). Detection of HPV-16 genome in human oral cancers and potentially malignant lesions from India. Oral Oncol..

[54] de Abreu P.M., Có A.C.G., Azevedo P.L., do Valle I.B., de Oliveira K.G., Gouvea S.A., Cordeiro-Silva M.F., Louro I.D., de Podestá J.R.V., Lenzi J. (2018). Frequency of HPV in oral cavity squamous cell carcinoma. BMC Cancer.

[55] de Menezes S.A.F., Miranda Y.M.S., da Silva Y.M., Carvalho T.R.B., Alves F.R.S., Silvestre R.V.D., Oliveira-Filho A.B., de Alencar Menezes T.O., de Souza Fonseca R.R., Laurentino R.V. (2022). Prevalence and Genotyping of HPV in Oral Squamous Cell Carcinoma in Northern Brazil. Pathogens.

[56] Ramos G.M.D., Cotter T.G., Ramos L.F., Floril V.T., Martinez G.A.R., Ruiz-Cabezas J.C. (2018). A pilot study on the identification of human papillomavirus genotypes in tongue cancer samples from a single institution in Ecuador. Braz. J. Med. Biol. Res. Rev. Bras. Pesqui. Medicas Biol..

[57] Dhanapal R., Ranganathan K., Kondaiah P., Devi R.U., Joshua E., Saraswathi T.R. (2015). High-risk human papilloma virus in archival tissues of oral pathosis and normal oral mucosa. Contemp. Clin. Dent..

[58] Dirasantchu S., Marthala M., Shaik S., Jayam R., Venkata S.S., Bokkasam V. (2015). Detection of human papilloma virus (HPV) and human immunodeficiency virus (HIV) in oral squamous cell carcinoma: A polymerized chain reaction (PCR) study. J. Indian Acad. Oral Med. Radiol..

[59] Duncan L.D., Winkler M., Carlson E.R., Heidel R.E., Kang E., Webb D. (2013). p16 immunohistochemistry can be used to detect human papillomavirus in oral cavity squamous cell carcinoma. J. Oral Maxillofac. Surg..

[60] Elango K.J., Suresh A., Erode E.M., Subhadradevi L., Ravindran H.K., Iyer S.K., Iyer S.K., Kuriakose M.A. (2011). Role of human papilloma virus in oral tongue squamous cell carcinoma. Asian Pac. J. Cancer Prev..

[61] Emmett S., Boros S., Whiteman D.C., Porceddu S.V., Panizza B.J., Antonsson A. (2018). Sexual behaviour, HPV status and p16(INK4a) expression in oropharyngeal and oral cavity squamous cell carcinomas: A case-case comparison study. J. Gen. Virol..

[62] Emmett S., Jenkins G., Boros S., Whiteman D.C., Panizza B., Antonsson A. (2017). Low prevalence of human papillomavirus in oral cavity squamous cell carcinoma in Queensland, Australia. ANZ J. Surg..

[63] Gan L.L., Zhang H., Guo J.H., Fan M.W. (2014). Prevalence of human papillomavirus infection in oral squamous cell carcinoma: A case-control study in Wuhan, China. Asian Pac. J. Cancer Prev..

[64] Giovannelli L., Campisi G., Colella G., Capra G., Di Liberto C., Caleca M.P., Matranga D., D’Angelo M., Lo Muzio L., Ammatuna P. (2006). Brushing of oral mucosa for diagnosis of HPV infection in patients with potentially malignant and malignant oral lesions. Mol. Diagn. Ther..

[65] González-Ramírez I., Irigoyen-Camacho M.E., Ramírez-Amador V., Lizano-Soberón M., Carrillo-García A., García-Carrancá A., Sánchez-Pérez Y., Méndez-Martínez R., Granados-García M., Ruíz-Godoy L. (2013). Association between age and high-risk human papilloma virus in Mexican oral cancer patients. Oral Dis..

[66] Goto M., Hanai N., Nishikawa D., Nagao T., Hasegawa Y. (2023). Prognosis of HPV-Positive Oral Squamous Carcinoma: A Cohort Study from Japan. J. Hard Tissue Biol..

[67] Götz C., Drecoll E., Straub M., Bissinger O., Wolff K.D., Kolk A. (2016). Impact of HPV infection on oral squamous cell carcinoma. Oncotarget.

[68] Grewal R.K., Sircar K., Bhat K.G., Grewal D.S., Tyagi K.K., David S. (2018). Detection of human papilloma virus-E6/E7 proteins of high-risk human papilloma virus in saliva and lesional tissue of oral squamous cell carcinoma patients using nested multiplex polymerase chain reaction: A comparative study. J. Oral Maxillofac. Pathol..

[69] Ha P.K., Pai S.I., Westra W.H., Gillison M.L., Tong B.C., Sidransky D., Califano J.A. (2002). Real-time quantitative PCR demonstrates low prevalence of human papillomavirus type 16 in premalignant and malignant lesions of the oral cavity. Clin. Cancer Res..

[70] Harbor S.N., Schneider J.W., Solomons N., Sanderson M., Afrogheh A.H. (2024). An Evaluation of High-Risk HPV in Squamous Cell Carcinomas of the Lip in a South African Cohort. Head Neck Pathol..

[71] Huang C.G., Lee L.A., Liao C.T., Yen T.C., Yang S.L., Liu Y.C., Li J.C., Gong Y.N., Kang C.J., Huang S.F. (2017). Molecular and serologic markers of HPV 16 infection are associated with local recurrence in patients with oral cavity squamous cell carcinoma. Oncotarget.

[72] Huang S.F., Li H.F., Liao C.T., Wang H.M., Chen I.H., Chang J.T., Chen Y.J., Cheng A.J. (2012). Association of HPV infections with second primary tumors in early-staged oral cavity cancer. Oral Dis..

[73] Ishibashi M., Kishino M., Sato S., Morii E., Ogawa Y., Aozasa K., Kogo M., Toyosawa S. (2011). The prevalence of human papillomavirus in oral premalignant lesions and squamous cell carcinoma in comparison to cervical lesions used as a positive control. Int. J. Clin. Oncol..

[74] Jaber L., Fatani H., Aldhahri S.F. (2019). Absence of human papillomavirus in oral cavity squamous cell carcinomas among Saudi patients. Clin. Exp. Dent. Res..

[75] Jalouli J., Ibrahim S.O., Mehrotra R., Jalouli M.M., Sapkota D., Larsson P.A., Hirsch J.M. (2010). Prevalence of viral (HPV, EBV, HSV) infections in oral submucous fibrosis and oral cancer from India. Acta Oto-Laryngol..

[76] Jalouli J., Jalouli M.M., Sapkota D., Ibrahim S.O., Larsson P.A., Sand L. (2012). Human papilloma virus, herpes simplex virus and epstein barr virus in oral squamous cell carcinoma from eight different countries. Anticancer Res..

[77] Jitani A.K., Raphael V., Mishra J., Shunyu N.B., Khonglah Y., Medhi J. (2015). Analysis of Human Papilloma Virus 16/18 DNA and its Correlation with p16 Expression in Oral Cavity Squamous Cell Carcinoma in North-Eastern India: A Chromogenic in-situ Hybridization Based Study. J. Clin. Diagn. Res..

[78] Kaminagakura E., Villa L.L., Andreoli M.A., Sobrinho J.S., Vartanian J.G., Soares F.A., Nishimoto I.N., Rocha R., Kowalski L.P. (2012). High-risk human papillomavirus in oral squamous cell carcinoma of young patients. Int. J. Cancer.

[79] Khanna R., Rao G.R., Tiwary S.K., Rai A., Khanna S., Khanna A.K. (2009). Detection of human papilloma virus 16 and 18 DNA sequences by southern blot hybridization in oral leukoplakia and squamous cell carcinoma. Indian J. Surg..

[80] Khovidhunkit S.O., Buajeeb W., Sanguansin S., Poomsawat S., Weerapradist W. (2008). Detection of human papillomavirus in oral squamous cell carcinoma, leukoplakia and lichen planus in Thai patients. Asian Pac. J. Cancer Prev..

[81] Kim S.M., Kwon I.J., Myoung H., Lee J.H., Lee S.K. (2018). Identification of human papillomavirus (HPV) subtype in oral cancer patients through microarray technology. Eur. Arch. Oto-Rhino-Laryngol..

[82] Klozar J., Kratochvil V., Salakova M., Smahelova J., Vesela E., Hamsikova E., Betka J., Tachezy R. (2008). HPV status and regional metastasis in the prognosis of oral and oropharyngeal cancer. Eur. Arch. Oto-Rhino-Laryngol..

[83] Kouketsu A., Sato I., Abe S., Oikawa M., Shimizu Y., Takahashi T., Kumamoto H. (2016). Detection of human papillomavirus infection in oral squamous cell carcinoma: A cohort study of Japanese patients. J. Oral Pathol. Med..

[84] Kulkarni S.S., Kulkarni S.S., Vastrad P.P., Kulkarni B.B., Markande A.R., Kadakol G.S., Hiremath S.V., Kaliwal S., Patil B.R., Gai P.B. (2011). Prevalence and distribution of high risk human papillomavirus (HPV) Types 16 and 18 in Carcinoma of cervix, saliva of patients with oral squamous cell carcinoma and in the general population in Karnataka, India. Asian Pac. J. Cancer Prev..

[85] Lee L.A., Huang C.G., Liao C.T., Lee L.Y., Hsueh C., Chen T.C., Lin C.Y., Fan K.H., Wang H.M., Huang S.F. (2012). Human papillomavirus-16 infection in advanced oral cavity cancer patients is related to an increased risk of distant metastases and poor survival. PLoS ONE.

[86] Lee L.A., Huang C.G., Tsao K.C., Liao C.T., Kang C.J., Chang K.P., Huang S.F., Chen I.H., Fang T.J., Li H.Y. (2015). Human Papillomavirus Infections are Common and Predict Mortality in a Retrospective Cohort Study of Taiwanese Patients with Oral Cavity Cancer. Medicine.

[87] Liang X.H., Lewis J., Foote R., Smith D., Kademani D. (2008). Prevalence and significance of human papillomavirus in oral tongue cancer: The Mayo Clinic experience. J. Oral Maxillofac. Surg..

[88] Lukesova E., Boucek J., Rotnaglova E., Salakova M., Koslabova E., Grega M., Eckschlager T., Rihova B., Prochazka B., Klozar J. (2014). High level of Tregs is a positive prognostic marker in patients with HPV-positive oral and oropharyngeal squamous cell carcinomas. BioMed Res. Int..

[89] Matzow T., Boysen M., Kalantari M., Johansson B., Hagmar B. (1998). Low detection rate of HPV in oral and laryngeal carcinomas. Acta Oncol..

[90] Montaldo C., Mastinu A., Zorco S., Santini N., Pisano E., Piras V., Denotti G., Peluffo C., Erriu M., Garau V. (2010). Distribution of human papillomavirus genotypes in sardinian patients with oral squamous cell carcinoma. Open Virol. J..

[91] More P., Kheur S., Patekar D., Kheur M., Gupta A.A., Raj A.T., Patil S. (2020). Assessing the nature of the association of human papillomavirus in oral cancer with and without known risk factors. Transl. Cancer Res..

[92] Nagpal J.K., Patnaik S., Das B.R. (2002). Prevalence of high-risk human papilloma virus types and its association with P53 codon 72 polymorphism in tobacco addicted oral squamous cell carcinoma (OSCC) patients of Eastern India. Int. J. Cancer.

[93] Naqvi S.U., Khan S., Ahmed A., Lail A., Gul S., Ahmed S. (2020). Prevalence of EBV, CMV, and HPV in oral squamous cell carcinoma patients in the Pakistani population. J. Med. Virol..

[94] Nauta I.H., Heideman D.A.M., Brink A., van der Steen B., Bloemena E., Koljenović S., Baatenburg de Jong R.J., Leemans C.R., Brakenhoff R.H. (2021). The unveiled reality of human papillomavirus as risk factor for oral cavity squamous cell carcinoma. Int. J. Cancer.

[95] Nola-Fuchs P., Boras V.V., Plecko V., Plestina S., Milenović A., Susić M., Brailo V. (2012). The prevalence of human papillomavirus 16 and Epstein-Barr virus in patients with oral squamous cell carcinoma. Acta Clin. Croat..

[96] Oliveira L.R., Ribeiro-Silva A., Ramalho L.N., Simões A.L., Zucoloto S. (2008). HPV infection in Brazilian oral squamous cell carcinomapatients and its correlation with clinicopathological outcomes. Mol. Med. Rep..

[97] Ostwald C., Rutsatz K., Schweder J., Schmidt W., Gundlach K., Barten M. (2003). Human papillomavirus 6/11, 16 and 18 in oral carcinomas and benign oral lesions. Med. Microbiol. Immunol..

[98] Palmieri A., Scapoli L., Martinelli M., Pezzetti F., Girardi A., Spinelli G., Lucchese A., Carinci F. (2011). Incidence of low risk human papillomavirus in oral cancer: A real time PCR study on 278 patients. Int. J. Immunopathol. Pharmacol..

[99] Panneerselvam K., Rameshkumar A., Rajkumar K., Ramadoss R. (2019). Detection of human papillomavirus 16 and 18 in patients with oral squamous cell carcinoma and potentially malignant oral disorders in South Indian population: A pilot study. J. Cancer Res. Ther..

[100] Panzarella V., Campisi G., Giardina Y., Maniscalco L., Capra G., Rodolico V., Di Fede O., Mauceri R. (2021). Low Frequency of Human Papillomavirus in Strictly Site-Coded Oral Squamous Cell Carcinomas, Using the Latest NHI/SEER-ICD Systems: A Pilot Observational Study and Critical Review. Cancers.

[101] Parshad S., Nandi S., Marwah N., Mehta P., Tripathi M., Netrapal, Gogna S., Karwasra R.K. (2015). Human papillomavirus 16 and 18 in squamous cell carcinoma of oral cavity and sexual practices: A pilot study at a Tertiary Care Hospital of North India. Natl. J. Maxillofac. Surg..

[102] Patel K.R., Vajaria B.N., Begum R., Desai A., Patel J.B., Shah F.D., Shukla S.N., Patel P.S. (2014). Prevalence of high-risk human papillomavirus type 16 and 18 in oral and cervical cancers in population from Gujarat, West India. J. Oral Pathol. Med..

[103] Petito G., Carneiro M.A., Santos S.H., Silva A.M., Alencar R.C., Gontijo A.P., Saddi V.A. (2017). Human papillomavirus in oral cavity and oropharynx carcinomas in the central region of Brazil. Braz. J. Otorhinolaryngol..

[104] Phusingha P., Ekalaksananan T., Vatanasapt P., Loyha K., Promthet S., Kongyingyoes B., Patarapadungkit N., Chuerduangphui J., Pientong C. (2017). Human papillomavirus (HPV) infection in a case-control study of oral squamous cell carcinoma and its increasing trend in northeastern Thailand. J. Med. Virol..

[105] Pintos V.L.J. (2002). Human Papillomavirus Infection and Oral Cancer: A Case-Control Study. Ph.D. Thesis.

[106] Polz D., Polz-Dacewicz M., Morshed K., Jędrych M. (2010). Prevalence of human papillomavirus in oral and oropharynx squamous cell carcinoma. Bull. Vet. Inst. Pulawy.

[107] Polz-Gruszka D., Morshed K., Stec A., Polz-Dacewicz M. (2015). Prevalence of Human papillomavirus (HPV) and Epstein-Barr virus (EBV) in oral and oropharyngeal squamous cell carcinoma in south-eastern Poland. Infect. Agents Cancer.

[108] Premoli-De-Percoco G., Ramirez J.L. (2001). High risk human papillomavirus in oral squamous carcinoma: Evidence of risk factors in a Venezuelan rural population. Preliminary report. J. Oral Pathol. Med..

[109] Purwanto D.J., Soedarsono N., Reuwpassa J.O., Adisasmita A.C., Ramli M., Djuwita R. (2020). The prevalence of oral high-risk HPV infection in Indonesian oral squamous cell carcinoma patients. Oral Dis..

[110] Rahbarnia L., Farajnia S., Bayazian G., Naderpour M., Feizi H. (2019). Prevalence of human papillomavirus in patients with oral squamous cell carcinoma in Tabriz, Iran. Crescent J. Med. Biol. Sci..

[111] Prakash S.M.R., Jha R.K., Chawla R., Kupendra S., Kamarthi N., Jugade S.C., Tiwari H.D. (2024). Assessment of the Impact of HPV Infection on the Incidence and Prognosis of Oral Cancers. J. Pharm. Bioallied Sci..

[112] Rivero E.R., Nunes F.D. (2006). HPV in oral squamous cell carcinomas of a Brazilian population: Amplification by PCR. Braz. Oral Res..

[113] Rodríguez-Santamarta T., Rodrigo J.P., García-Pedrero J.M., Álvarez-Teijeiro S., Ángeles Villaronga M., Suárez-Fernández L., Alvarez-Argüelles M.E., Astudillo A., de Vicente J.C. (2016). Prevalence of human papillomavirus in oral squamous cell carcinomas in northern Spain. Eur. Arch. Oto-Rhino-Laryngol..

[114] Rout T., Panda S.K., Shankar K.V., Kar D., Mohanty D.P., Agrawala S. (2024). Prevalence of HPV in Oral Squamous Cell Carcinoma Through p16 IHC: A Hospital-Based Study in Eastern India. Niger. J. Basic Clin. Sci..

[115] Rungraungrayabkul D., Panpradit N., Lapthanasupkul P., Kitkumthorn N., Klanrit P., Subarnbhesaj A., Sresumatchai V., Klongnoi B., Khovidhunkit S.P. (2022). Detection of Human Papillomavirus and p16(INK4a) Expression in Thai Patients with Oral Squamous Cell Carcinoma. Head Neck Pathol..

[116] Saini R., Tang T.H., Zain R.B., Cheong S.C., Musa K.I., Saini D., Ismail A.R., Abraham M.T., Mustafa W.M., Santhanam J. (2011). Significant association of high-risk human papillomavirus (HPV) but not of p53 polymorphisms with oral squamous cell carcinomas in Malaysia. J. Cancer Res. Clin. Oncol..

[117] Schwartz S.R., Yueh B., McDougall J.K., Daling J.R., Schwartz S.M. (2001). Human papillomavirus infection and survival in oral squamous cell cancer: A population-based study. Otolaryngol.—Head Neck Surg..

[118] Shima K., Kobayashi I., Saito I., Kiyoshima T., Matsuo K., Ozeki S., Ohishi M., Sakai H. (2000). Incidence of human papillomavirus 16 and 18 infection and p53 mutation in patients with oral squamous cell carcinoma in Japan. Br. J. Oral Maxillofac. Surg..

[119] Sichero L., Gonçalves M.G., Bettoni F., Coser E.M., Mota G., Nunes R.A.L., Mercante A., Natalino R., Uno M., Ferreira Alves M.J. (2024). Detection of serum biomarkers of HPV-16 driven oropharynx and oral cavity cancer in Brazil. Oral Oncol..

[120] Simonato L.E., Garcia J.F., Sundefeld M.L., Mattar N.J., Veronese L.A., Miyahara G.I. (2008). Detection of HPV in mouth floor squamous cell carcinoma and its correlation with clinicopathologic variables, risk factors and survival. J. Oral Pathol. Med..

[121] Singh A.K., Kushwaha J.K., Anand A., Sonkar A.A., Husain N., Srivastava K., Singh S. (2016). Human Papilloma Virus in Oral Cavity Cancer and Relation to Change in Quality of Life Following Treatment-a Pilot Study from Northern India. Indian J. Surg. Oncol..

[122] Singh V., Husain N., Akhtar N., Kumar V., Tewari S., Mishra S., Misra S., Khan M.Y. (2015). Do Human Papilloma Viruses Play Any Role in Oral Squamous Cell Carcinoma in North Indians?. Asian Pac. J. Cancer Prev..

[123] Smith E.M., Hoffman H.T., Summersgill K.S., Kirchner H.L., Turek L.P., Haugen T.H. (1998). Human papillomavirus and risk of oral cancer. Laryngoscope.

[124] Soares R.C., Oliveira M.C., Souza L.B., Costa A.L., Medeiros S.R., Pinto L.P. (2007). Human papillomavirus in oral squamous cells carcinoma in a population of 75 Brazilian patients. Am. J. Otolaryngol..

[125] Sri S., Ramani P., Premkumar P., Ramshankar V., Ramasubramanian A., Krishnan R.P. (2021). Prevalence of Human Papillomavirus (HPV) 16 and 18 in Oral Malignant and Potentially Malignant Disorders: A Polymerase Chain Reaction Analysis—A Comparative Study. Ann. Maxillofac. Surg..

[126] Taberna M., Inglehart R.C., Pickard R.K., Fakhry C., Agrawal A., Katz M.L., Gillison M.L. (2017). Significant changes in sexual behavior after a diagnosis of human papillomavirus-positive and human papillomavirus-negative oral cancer. Cancer.

[127] Tachezy R., Klozar J., Saláková M., Smith E., Turek L., Betka J., Kodet R., Hamsíková E. (2005). HPV and other risk factors of oral cavity/oropharyngeal cancer in the Czech Republic. Oral Dis..

[128] Tang K.D., Menezes L., Baeten K., Walsh L.J., Whitfield B.C.S., Batstone M.D., Kenny L., Frazer I.H., Scheper G.C., Punyadeera C. (2020). Oral HPV16 Prevalence in Oral Potentially Malignant Disorders and Oral Cavity Cancers. Biomolecules.

[129] Tangthongkum M., Phisalmongkhon S., Leelasawatsuk P., Supanimitjaroenporn P., Kirtsreesakul V., Tantipisit J. (2024). Impact of human papillomavirus status on survival in patients with oral cancer. Laryngoscope Investig. Otolaryngol..

[130] Tealab S.H., Sedhom N.F.H., Hassouna A., Gouda I., Ismail H. (2019). Prevalence of human papilloma virus in oropharyngeal, tongue and lip squamous cell carcinoma: An experience from the Egyptian National Cancer Institute. J. Investig. Med..

[131] Tokuzen N., Nakashiro K.I., Tojo S., Goda H., Kuribayashi N., Uchida D. (2021). Human papillomavirus-16 infection and p16 expression in oral squamous cell carcinoma. Oncol. Lett..

[132] Tsimplaki E., Argyri E., Xesfyngi D., Daskalopoulou D., Stravopodis D.J., Panotopoulou E. (2014). Prevalence and expression of human papillomavirus in 53 patients with oral tongue squamous cell carcinoma. Anticancer Res..

[133] Valls-Ontañón A., Hernández-Losa J., Somoza Lopez de Haro R., Bellosillo-Paricio B., Ramón Y.C.S., Bescós-Atín C., Munill-Ferrer M., Alberola-Ferranti M. (2019). Impact of human papilloma virus in patients with oral and oropharyngeal squamous cell carcinomas. Med. Clin..

[134] Vanshika S., Preeti A., Sumaira Q., Vijay K., Shikha T., Shivanjali R., Shankar S.U., Mati G.M. (2021). Incidence OF HPV and EBV in oral cancer and their clinico-pathological correlation- a pilot study of 108 cases. J. Oral Biol. Craniofacial Res..

[135] Verma H., Singh S.K., Phulambrikar T., Gupta A. (2018). Evaluation of human papillomavirus as an independent risk factor in known patients of oral squamous cell carcinoma using immunohistochemistry. J. Indian Acad. Oral Med. Radiol..

[136] Loustau A.C.V., Dulguerov N., Curvoisier D., McKee T., Lombardi T. (2019). Low prevalence of HPV-induced oral squamous cell carcinoma in Geneva, Switzerland. Oral Dis..

[137] Yang L.Q., Xiao X., Li C.X., Wu W.Y., Shen X.M., Zhou Z.T., Fan Y., Shi L.J. (2019). Human papillomavirus genotypes and p16 expression in oral leukoplakia and squamous cell carcinoma. Int. J. Clin. Exp. Pathol..

[138] Zhang Z.Y., Sdek P., Cao J., Chen W.T. (2004). Human papillomavirus type 16 and 18 DNA in oral squamous cell carcinoma and normal mucosa. Int. J. Oral Maxillofac. Surg..

[139] Christianto S., Li K.Y., Huang T.H., Su Y.X. (2022). The Prognostic Value of Human Papilloma Virus Infection in Oral Cavity Squamous Cell Carcinoma: A Meta-Analysis. Laryngoscope.

[140] Lima M.A.P.d., Silva C.G.L.d., Rabenhorst S.H.B. (2014). Association between human papillomavirus (HPV) and the oral squamous cell carcinoma: A systematic review. J. Bras. Patol. Med. Lab..

[141] Shigeishi H., Sugiyama M. (2016). Risk factors for oral human papillomavirus infection in healthy individuals: A systematic review and meta-analysis. J. Clin. Med. Res..

[142] Kaur G., Yap T., Ramani R., McCullough M., Singh A. (2024). Assessing bias in the causal role of HPV in oral cancer: A systematic review and meta-analysis. Oral Dis..

[143] Sathish N., Wang X., Yuan Y. (2014). Human papillomavirus (HPV)-associated oral cancers and treatment strategies. J. Dent. Res..

[144] Shaikh M.H., McMillan N.A., Johnson N.W. (2015). HPV-associated head and neck cancers in the Asia Pacific: A critical literature review & meta-analysis. Cancer Epidemiol..

[145] Shavers V.L., Harlan L.C., Winn D., Davis W.W. (2003). Racial/ethnic patterns of care for cancers of the oral cavity, pharynx, larynx, sinuses, and salivary glands. Cancer Metastasis Rev..

