# Supplementary material for: Global Prevalence and Modifiers of Human Papillomavirus Positivity in Oral Cavity Cancer: A Systematic Review and Meta-Analysis of Prevalence (1995–2024)
Source: Cancers (Basel). 2025 Aug 31;17(17):2870. doi: 10.3390/cancers17172870 (PMC12427480; doi:10.3390/cancers17172870)
Supplement: Supplementary file 1 [file cancers-17-02870-s001.zip › cancers-3836138-supplementary.pdf]

Table S1. The detailed search criteria employed in the literature search [date of search: October 9th, 2024]

Database	No.	Search Query	Results
**PubMed**	#1	HPV[tiab] OR "human papillomavirus"[tiab] OR "Human Papillomavirus Viruses"[Mesh]	65945
	#2	“oral cancer”[tiab] OR “oral carcinoma”[tiab] OR “oral neoplasm”[tiab] OR “oral cavity cancer”[tiab] OR “oral cavity carcinoma”[tiab] OR “oral cavity neoplasm”[tiab] OR “mouth cancer”[tiab] OR “mouth carcinoma”[tiab] OR “mouth neoplasm”[tiab] OR “gingival cancer”[tiab] OR “gingival carcinoma”[tiab] OR “gingival neoplasm”[tiab] OR “lip cancer”[tiab] OR “lip carcinoma”[tiab] OR “lip neoplasm”[tiab] OR “tongue cancer”[tiab] OR “tongue carcinoma”[tiab] OR “tongue neoplasm”[tiab] OR “buccal cancer”[tiab] OR “buccal carcinoma”[tiab] OR “palate cancer”[tiab] OR “palate carcinoma”[tiab] OR “palate neoplasm”[tiab] OR “oral cavity squamous”[tiab] OR “oral squamous”[tiab] OR "Mouth Neoplasms"[Mesh]	95385
	#3	Prevalence[tiab] OR prevalent[tiab] OR frequency[tiab] OR rate[tiab] OR incidence[tiab] OR proportion[tiab] OR occurrence[tiab] OR “cross-sectional”[tiab]	6096303
	#4	#1 AND #2 AND #3	2082
**Scopus**	#1	TITLE-ABS-KEY (HPV) OR TITLE-ABS-KEY ("human papillomavirus")	79352
	#2	TITLE-ABS-KEY (“oral cancer”) OR TITLE-ABS-KEY (“oral carcinoma”) OR TITLE-ABS-KEY (“oral neoplasm”) OR TITLE-ABS-KEY (“oral cavity cancer”) OR TITLE-ABS-KEY (“oral cavity carcinoma”) OR TITLE-ABS-KEY (“oral cavity neoplasm”) OR TITLE-ABS-KEY (“mouth cancer”) OR TITLE-ABS-KEY (“mouth carcinoma”) OR TITLE-ABS-KEY (“mouth neoplasm”) OR TITLE-ABS-KEY (“gingival cancer”) OR TITLE-ABS-KEY (“gingival carcinoma”) OR TITLE-ABS-KEY (“gingival neoplasm”) OR TITLE-ABS-KEY (“lip cancer”) OR TITLE-ABS-KEY (“lip carcinoma”) OR TITLE-ABS-KEY (“lip neoplasm”) OR TITLE-ABS-KEY (“tongue cancer”) OR TITLE-ABS-KEY (“tongue carcinoma”) OR TITLE-ABS-KEY (“tongue neoplasm”) OR TITLE-ABS-KEY (“buccal cancer”) OR TITLE-ABS-KEY (“buccal carcinoma”) OR TITLE-ABS-KEY (“palate cancer”) OR TITLE-ABS-KEY (“palate carcinoma”) OR TITLE-ABS-KEY (“palate neoplasm”) OR TITLE-ABS-KEY (“oral cavity squamous”) OR TITLE-ABS-KEY (“oral squamous”)	93759
	#3	TITLE-ABS-KEY (Prevalence) OR TITLE-ABS-KEY (prevalent) OR TITLE-ABS-KEY (frequency) OR TITLE-ABS-KEY (rate) OR TITLE-ABS-KEY (incidence) OR TITLE-ABS-KEY (proportion) OR TITLE-ABS-KEY (occurrence) OR TITLE-ABS-KEY (“cross-sectional”)	16919615
	#4	#1 AND #2 AND #3	2757
**Web of Science**	#1	AB=HPV OR AB="human papillomavirus"	56074
	#2	AB=“oral cancer” OR AB=“oral carcinoma” OR AB=“oral neoplasm” OR AB=“oral cavity cancer” OR AB=“oral cavity carcinoma” OR AB=“oral cavity neoplasm” OR AB=“mouth cancer” OR AB=“mouth carcinoma” OR AB="mouth neoplasm” OR AB=“gingival cancer” OR AB=“gingival carcinoma” OR AB=“gingival neoplasm” OR AB=“lip cancer” OR AB=“lip carcinoma” OR AB=“lip neoplasm” OR AB=“tongue cancer” OR AB=“tongue carcinoma” OR AB=“tongue neoplasm” OR AB=“buccal cancer” OR	31876

		AB="buccal carcinoma" OR AB= "palate cancer" OR AB="palate carcinoma" OR AB="palate neoplasm" OR AB="oral cavity squamous" OR AB= "oral squamous"	
	#3	AB=Prevalence OR AB=prevalent OR AB=frequency OR AB=rate OR AB=incidence OR AB=proportion OR AB=occurrence OR AB="cross-sectional"	11811551
	#4	#1 AND #2 AND #3	1131
**Google Scholar**	With all of the words	Oral cavity cancer	-
	With the exact phrase	human papillomavirus	-
	With at least one of the words	Prevalence incidence frequency rate	-
	Total	Only the first 200 records were retrieved as per recent recommendations	200

Table S2. A summary of the methodological quality of included studies using the National Institute of Health quality assessment tool

Author (YOP	Q1	Q2	Q3	Q4	Q5	Q6	Q7	Q8	Q9	Q10	Q11	Q12	Q13	Q14	Total Score	Overall Grade
Abreu (2018)	2	1	1	2	0	2	2	2	2	1	2	1	1	0	19	Fair
Abreu (2020)	2	1	0	1	0	2	2	2	2	2	2	1	0	0	17	Fair
ADAMOPOULOU (2008)	2	1	0	1	0	2	2	0	2	1	1	1	0	0	13	Fair
Adilbay (2018)	1	1	0	1	0	2	2	1	2	2	2	1	0	0	15	Fair
Afzal (2019)	2	1	2	2	0	2	2	2	2	2	2	1	2	0	22	Good
Ahmed (2019)	1	1	0	2	0	2	2	1	2	1	2	1	0	0	15	Fair
Ajila (2021)	2	1	2	1	0	1	2	2	2	1	2	0	2	0	18	Fair
Akhondnezhad (2018)	2	1	0	0	0	2	2	1	2	1	2	1	0	0	14	Fair
Ali (2008)	2	1	2	2	0	1	2	1	2	2	2	1	2	0	20	Good
Alsharif (2021)	2	1	2	2	0	2	2	2	2	2	2	1	2	0	22	Good
Ana (2019)	2	1	2	2	0	1	2	1	2	1	2	1	2	0	19	Fair
Antuncov (2022)	2	1	2	2	0	2	2	1	2	1	2	1	0	0	18	Fair
Anwar (2024)	1	1	0	1	0	2	2	1	2	1	2	1	0	0	14	Fair
Ashraf (2017)	1	1	2	1	0	1	2	2	2	2	2	0	2	0	18	Fair
Balaram (1995)	2	1	0	2	0	2	2	2	2	1	2	1	2	0	19	Fair
Belobrov (2017)	2	1	0	1	0	2	2	2	2	2	2	1	0	0	17	Fair
Bijina (2020)	2	1	0	0	0	1	2	2	2	1	2	1	2	0	16	Fair
Boy (2006)	1	1	0	1	0	2	2	1	2	1	2	1	2	0	16	Fair
Božić (2020)	2	1	2	1	0	2	2	1	2	1	2	1	2	0	19	Fair
Campisi (2006)	2	1	0	0	0	2	2	2	2	2	2	1	2	0	18	Fair
Chakrobarty (2014)	2	1	2	2	0	1	2	2	2	2	2	1	2	0	21	Good
Chen (2012)	2	1	2	1	0	2	2	2	2	1	2	1	1	0	19	Fair
Chen (2016)	2	1	0	0	0	2	2	2	2	1	2	1	0	0	15	Fair
Chotipanich (2018)	2	1	2	1	0	1	2	2	2	2	2	1	2	0	20	Good
Chowdary (2018)	1	1	0	1	0	1	2	2	2	1	2	1	2	0	16	Fair
Cutilli (2016)	2	1	0	1	0	1	2	1	2	1	2	1	2	0	16	Fair
DAHLGREN (2004)	2	1	0	1	0	1	2	1	2	1	2	1	2	0	16	Fair
D'Costa (1998)	2	1	2	1	0	2	2	2	2	2	2	1	2	0	21	Good
Dhanapal (2015)	2	1	0	1	0	1	2	1	2	1	2	1	2	0	16	Fair

Duncan (2013)	2	1	2	1	2	2	2	1	2	1	2	1	2	0	21	Good
Elango (2011)	2	1	2	1	0	1	2	2	2	2	2	1	2	0	20	Good
Emmett (2017)	2	1	0	2	0	2	2	2	2	2	2	1	0	0	18	Fair
Emmett (2018)	2	1	2	2	0	2	2	2	2	2	2	1	2	0	22	Good
Fuchs (2012)	2	1	2	1	0	1	2	1	2	1	1	1	2	0	17	Fair
Gan (2014)	1	1	2	1	0	1	2	0	2	1	2	1	2	0	16	Fair
Giovannelli (2006)	2	1	0	1	0	2	2	1	2	1	2	1	0	0	15	Fair
Goto (2023)	1	1	0	1	0	2	2	2	2	2	2	1	2	0	18	Fair
Götz (2016)	2	1	0	0	0	2	2	1	2	1	2	1	0	0	14	Fair
Ha (2022)	2	1	0	2	0	2	2	1	2	1	2	1	0	0	16	Fair
Harbor (2024)	2	1	0	1	0	1	2	1	2	1	2	1	2	0	16	Fair
Huang (2012)	2	1	0	1	0	2	2	1	2	1	2	1	0	0	15	Fair
Huang (2017)	2	1	1	1	0	2	2	2	2	2	2	1	2	0	20	Good
Ibieta (2005)	2	1	0	1	0	2	2	2	2	2	2	1	2	0	19	Fair
Ishibashi (2011)	2	1	2	1	0	2	2	1	2	1	2	1	2	0	19	Fair
Jaber (2019)	2	1	2	2	0	1	2	1	2	1	2	1	2	0	19	Fair
JALOULI (2010)	2	1	0	1	2	2	2	1	2	1	2	1	0	0	17	Fair
Jalouli (2012)	2	1	0	0	0	2	2	1	2	1	2	1	0	0	14	Fair
JitAni (2015)	2	1	2	1	0	2	2	1	2	1	2	1	2	0	19	Fair
Kaminagakura (2012)	2	1	1	1	0	1	2	2	2	2	1	1	2	0	18	Fair
KANSKY (2003)	2	1	0	1	0	1	2	2	2	2	2	1	2	0	18	Fair
Kaur (2018)	2	1	0	2	0	2	2	1	2	1	2	1	0	0	16	Fair
Khanna (2009)	1	1	0	1	0	1	2	2	2	2	2	1	2	0	17	Fair
Khovidhunkit (2008)	2	1	0	2	0	2	2	1	2	1	2	1	0	0	16	Fair
Kim (2018)	2	1	2	0	0	2	2	2	2	1	2	1	2	1	20	Good
Klozar (2008)	2	1	2	2	0	2	2	2	2	2	2	1	2	0	22	Good
Komolmala (2020)	2	1	0	1	0	2	2	1	2	1	2	1	0	0	15	Fair
Kouketsu (2015)	2	1	2	1	0	2	2	1	2	1	2	1	2	0	19	Fair
Kulkarni (2011)	2	1	0	2	0	2	2	1	2	1	2	1	0	0	16	Fair
Kumar (2007)	2	1	0	1	0	2	2	1	2	1	2	1	2	0	17	Fair
Lee (2012)	1	1	0	1	0	2	2	1	2	2	2	1	0	0	15	Fair

Lee (2015)	2	1	0	1	0	1	2	2	2	1	2	1	0	0	15	Fair
Liang (2008)	2	1	0	1	0	2	2	1	2	1	2	1	2	0	17	Fair
Lukesova (2014)	2	1	0	1	0	2	2	2	2	2	2	1	2	0	19	Fair
Machado (2010)	2	1	2	1	0	1	2	2	2	2	2	1	2	0	20	Good
Makvandi (2022)	2	1	1	1	0	2	2	2	2	2	2	1	1	0	19	Fair
Matzow (2009)	2	1	2	1	0	2	2	1	2	1	2	1	2	0	19	Fair
Menezes (2022)	2	1	2	2	0	2	2	1	2	2	2	1	2	0	21	Good
Montaldo (2010)	2	1	2	1	0	1	2	1	2	1	1	1	2	0	17	Fair
More (2020)	2	1	1	1	0	2	2	1	2	1	2	1	1	0	17	Fair
NAGPAL (2001)	2	1	2	1	0	1	2	2	2	2	1	1	0	0	17	Fair
Naqvi (2020)	2	1	0	0	0	2	2	1	2	1	2	1	0	0	14	Fair
Nauta (2021)	2	1	1	2	0	1	2	2	2	2	2	1	1	0	19	Fair
Nekić (2022)	2	1	0	1	0	1	2	2	2	1	2	1	0	0	15	Fair
OLIVEIRA (2003)	2	1	2	1	0	1	2	2	2	2	2	1	0	0	18	Fair
Ostwald (2003)	2	1	1	1	0	2	2	1	2	1	2	1	1	0	17	Fair
PALMIER (2011)	2	1	0	1	0	1	2	1	2	1	2	1	2	0	16	Fair
Panneerselvam (2019)	2	1	0	1	0	2	2	1	2	1	2	1	0	0	15	Fair
Panzarella (2021)	2	1	2	1	2	2	2	2	2	2	2	1	2	0	23	Good
Parshad (2015)	2	1	2	0	0	1	2	1	2	1	1	1	2	0	16	Fair
Patel (2015)	2	1	0	2	0	2	2	2	2	2	2	1	2	0	20	Good
Percoco (2001)	2	1	0	1	0	1	2	1	2	1	2	1	2	0	16	Fair
Petitoa (2017)	2	1	0	1	0	2	2	1	2	1	2	1	2	0	17	Fair
Petrovic (2023)	2	1	0	1	0	2	2	1	2	2	2	1	2	0	18	Fair
Phusingha (2016)	2	1	0	1	0	1	2	2	2	2	1	1	0	0	15	Fair
POLZ (2010)	2	1	0	1	0	2	2	2	2	2	2	1	0	0	17	Fair
Polz-Gruszka (2015)	2	1	2	1	0	1	2	2	2	2	2	1	2	0	20	Good
Pongsapich (2016)	2	1	2	1	2	2	2	2	2	2	2	1	2	0	23	Good
Prakash (2024)	2	1	2	1	0	1	2	1	2	1	2	1	0	0	16	Fair
Purwanto (2019)	2	1	0	1	0	1	2	1	2	1	2	1	2	0	16	Fair
Rahbarnia (2019)	1	1	2	0	0	1	2	1	2	1	1	1	2	0	15	Fair
Ramírez (2013)	2	1	2	1	0	1	2	2	2	2	2	1	2	0	20	Good

Ramos (2018)	2	1	0	1	0	2	2	1	2	1	2	1	2	0	17	Fair
Rivro (2006)	2	1	0	1	0	2	2	1	2	1	2	1	2	0	17	Fair
Rodrı´guez-Santamarta (2016)	2	1	2	1	0	1	2	1	2	1	2	1	2	0	18	Fair
ROMANITAN (2008)	2	1	2	1	0	2	2	1	2	1	2	1	0	0	17	Fair
Rout (2024)	2	1	0	1	0	2	2	1	2	1	2	1	2	0	17	Fair
Rungraungrayabkul (2022)	2	1	2	2	0	1	2	2	2	2	2	1	2	0	21	Good
Saini (2010)	2	1	2	1	0	1	2	2	2	2	1	1	2	0	19	Fair
Schwartz (2001)	2	1	0	1	0	2	2	1	2	1	2	1	0	0	15	Fair
Shima (2000)	2	1	2	1	0	2	2	2	2	2	2	1	2	0	21	Good
Sichero (2024)	2	1	0	1	0	2	2	2	2	2	2	1	2	0	19	Fair
Simonato (2008)	2	1	0	1	0	2	2	2	2	2	2	1	2	0	19	Fair
Singh (2015)	2	1	2	1	0	2	2	2	2	2	2	1	2	0	21	Good
Singh (2016)	2	1	2	0	0	2	2	2	2	2	2	1	2	0	20	Good
Smith (1998)	2	1	0	1	0	1	2	2	2	2	1	1	2	0	17	Fair
Soares (2007)	2	1	0	1	0	1	2	1	2	1	2	1	2	0	16	Fair
Sri (2021)	2	1	2	1	0	2	2	1	2	1	2	1	0	0	17	Fair
Suresh (2015)	2	1	2	1	0	1	2	2	2	2	2	1	2	0	20	Good
Taberna (2017)	1	1	0	0	0	1	2	2	2	2	2	1	2	0	16	Fair
Tachezy (2005)	1	1	1	2	0	2	2	1	2	1	2	1	0	0	16	Fair
Tang (2020)	2	1	2	1	0	2	2	2	2	2	2	1	2	0	21	Good
Tangthongkum (2024)	2	1	0	1	0	1	2	2	2	2	2	1	2	0	18	Fair
Tealab (2009)	2	1	2	1	0	1	2	2	2	2	2	1	2	0	20	Good
Tokuzen (2021)	1	1	2	1	0	2	2	2	2	2	2	1	2	0	20	Good
Torre (2018)	2	1	2	2	0	2	2	2	2	2	2	1	2	0	22	Good
TSIMPLAKI (2014)	2	1	0	1	0	2	2	1	2	1	2	1	0	0	15	Fair
Valls-Ontanón (2007)	2	1	2	2	0	1	2	2	2	2	2	1	2	0	21	Good
Vanshika (2021)	2	1	2	1	0	2	2	1	2	1	2	1	2	0	19	Fair
Vega (2002)	2	1	2	2	0	1	2	2	2	1	2	1	2	0	20	Good
Verma (2018)	2	1	2	0	0	1	2	1	2	1	1	1	2	0	16	Fair
Yang (2019)	2	1	2	0	0	1	2	2	2	2	1	1	2	0	18	Fair
Zhang (2004)	2	1	2	0	0	1	2	2	2	2	1	1	2	0	18	Fair

YOP: year of publication; an overall rating of good, fair, and poor was given for overall scores >20, 11-20, and <10, respectively.

Q1: Was the research question or objective in this paper clearly stated?
Q2: Was the study population clearly specified and defined?
Q3: Was the participation rate of eligible persons at least 50%?
Q4: Were all the subjects selected or recruited from the same or similar populations (including the same time period)? Were inclusion and exclusion criteria for being in the study prespecified and applied uniformly to all participants?
Q5: Was a sample size justification, power description, or variance and effect estimates provided?
Q6: For the analyses in this paper, were the exposure(s) of interest measured prior to the outcome(s) being measured?
Q7: Was the timeframe sufficient so that one could reasonably expect to see an association between exposure and outcome if it existed?
Q8: For exposures that can vary in amount or level, did the study examine different levels of the exposure as related to the outcome (e.g., categories of exposure, or exposure measured as continuous variable)?
Q9: Were the exposure measures (independent variables) clearly defined, valid, reliable, and implemented consistently across all study participants?
Q10: Was the exposure(s) assessed more than once over time?
Q11: Were the outcome measures (dependent variables) clearly defined, valid, reliable, and implemented consistently across all study participants?
Q12: Were the outcome assessors blinded to the exposure status of participants?
Q13: Was loss to follow-up after baseline 20% or less?
Q14: Were key potential confounding variables measured and adjusted statistically for their impact on the relationship between exposure(s) and outcome(s)?

Table S3. The prevalence of total HPV positivity in oral cancer patients stratified by the year of investigation

Year	Prevalence	95% Confidence Interval	Studies	Q	P-value	tau2	I^2 (%)	H2
**1995**	73.6	64.6-82.7	1	0	.	0	.	.
**1998**	15	1-20.1	2	0	0.992	0	0	1
**2000**	68	55.1-80.9	1	0	.	0	.	.
**2001**	35.8	10.9-60.8	3	44.13	0	0.046	96.23	26.52
**2002**	27.8	17.4-38.1	1	0	.	0	.	.
**2003**	23.6	3.6-43.7	3	35.79	0	0.03	94.57	18.4
**2004**	38	0-84	2	176.41	0	0.255	99.43	176.41
**2005**	47.4	38.2-56.6	2	1.05	0.306	0	4.73	1.05
**2006**	18.9	1.8-35.9	4	41.58	0	0.026	93.11	14.51
**2007**	21.6	9-34.2	3	8.59	0.014	0.009	77.9	4.53
**2008**	26	4.6-47.5	7	381.63	0	0.082	98.88	89.33
**2009**	33.3	0-67.7	3	72.53	0	0.09	97.51	40.2
**2010**	34.3	16.6-52	5	52.3	0	0.038	92.79	13.88
**2011**	34.9	2.2-67.6	4	168.42	0	0.109	99.1	111.13
**2012**	31.3	24.8-37.8	6	17.72	0.003	0.004	65.44	2.89
**2013**	6.4	2.6-10.1	2	0.85	0.358	0	0.05	1
**2014**	33.2	15.9-50.6	4	36.73	0	0.029	93.16	14.61
**2015**	19.9	11.4-28.5	9	246.22	0	0.014	96.85	31.71
**2016**	21.9	0-44.5	7	502	0	0.092	99.28	138.97
**2017**	17.4	4.1-30.7	6	65.68	0	0.025	95.53	22.36
**2018**	18.5	9.4-27.6	11	94	0	0.021	94.28	17.48
**2019**	31.4	9-53.8	8	384.7	0	0.1	98.41	62.86
**2020**	17.8	5.5-30.1	7	254.08	0	0.025	97.01	33.44
**2021**	10.5	5.7-15.3	7	49.59	0	0.003	82.2	5.62
**2022**	17.3	8.9-25.6	6	30.4	0	0.009	83.66	6.12
**2023**	13	6.1-20	2	0.39	0.53	0	0.02	1
**2024**	7.5	1.9-13.1	5	38.6	0	0.003	92.59	13.5
**Overall**	24.1	20.4-27.8	120	3832.06	0	0.041	98.48	65.66

Table S4. The prevalence of total HPV positivity in oral cancer patients stratified by the country of investigation

Country	Prevalence	95% Confidence Interval	Studies	Q	P-value	tau2	I^2 (%)	H2
**Australia**	24.1	3-45.2	4	55.2	0.000	0.044	95.98	24.85
**Brazil**	15.8	7.5-24	9	90.39	0.000	0.014	95.12	20.49
**Canada**	23.5	16.7-30.4	2	1.11	0.292	0	10.04	1.11
**China**	21.4	0-48.5	5	230.5	0.000	0.095	99.29	140.99
**Croatia**	35.6	17.2-54.1	2	2	0.158	0.009	49.91	2
**Czech Republic**	55.7	46.5-65	3	3.79	0.151	0.003	47.42	1.9
**Ecuador**	41.5	28.2-54.8	1	0	.	0	.	.
**Egypt**	33.3	24-42.6	1	0	.	0	.	.
**Germany**	20.9	3.3-38.4	4	87.47	0.000	0.031	98.13	53.56
**Greece**	5.4	0-15.3	2	4.77	0.029	0.004	79.05	4.77
**India**	30.4	21.9-38.9	23	546.91	0.000	0.039	96.89	32.18
**Indonesia**	17.9	9.4-26.5	1	0	.	0	.	.
**Iran**	13.9	8.6-19.2	4	5.51	0.138	0.001	46.11	1.86
**Iraq**	20	7.6-32.4	1	0	.	0	.	.
**Italy**	38.5	12.9-64.2	6	486.15	0.000	0.099	98.3	58.98
**Japan**	23.9	5.3-42.6	6	78.19	0.000	0.051	97.43	38.87
**Kazakhstan**	14.6	3.8-25.5	1	0	.	0	.	.
**Malaysia**	51.4	41.9-61	1	0	.	0	.	.
**Mexico**	22.9	0-59.2	2	25.05	0.000	0.066	96.01	25.05
**Montenegro**	23.3	12.6-34	1	0	.	0	.	.
**Pakistan**	28.1	0-59.5	4	335.6	0.000	0.101	99.62	260.35
**Poland**	21.8	10.6-32.9	2	1.76	0.185	0.003	43.08	1.76
**Saudi Arabia**	52.4	0-99.4	2	107.04	0.000	0.3	99.07	107.04
**Serbia**	15.8	6.8-24.9	2	0.03	0.870	0	0.01	1
**Singapore**	73.6	64.6-82.7	1	0	.	0	.	.
**Slovenia**	8.5	1.4-15.6	1	0	.	0	.	.
**South Africa**	16.1	6.3-25.9	2	1.97	0.160	0.003	49.34	1.97
**South Korea**	7	3.3-10.6	1	0	.	0	.	.

**Spain**	11.8	8-15.5	2	0.08	0.784	0	0.07	1
**Sweden**	13.3	0-34.1	3	62.11	0.000	0.033	97.6	41.67
**Switzerland**	7.7	3.5-11.9	1	0	.	0	.	.
**Taiwan**	22.9	15.2-30.7	5	19.12	0.001	0.006	89.67	9.68
**Thailand**	16.6	5.9-27.3	7	165.52	0.000	0.02	95.74	23.47
**The Netherlands**	4.3	0-9.4	2	4.14	0.042	0.001	75.86	4.14
**United Kingdom**	8.1	2.7-13.4	1	0	.	0	.	.
**United States of America**	9.8	2.7-16.9	4	43.21	0.000	0.005	89.68	9.69
**Venezuela**	60	46.4-73.6	1	0	.	0	.	.
**Overall**	24.1	20.4-27.8	120	3832.06	0.000	0.041	98.48	65.66

Table S5. Year-specific differences in HPV-16 and HPV-18 positivity in oral cavity cancer patients

	HPV-16								HPV-18							
	Prevalence	95% CI	Studies	Q	P	Tau^2	I^2	H2	Prevalence	95% CI	Studies	Q	P	Tau^2	I^2	H2
**1995**	41.8	31.6-51.9	1	0	.	0	.	.	47.3	37-57.5	1	0	.	0	.	.
**1998**	26	0-52.7	2	4.13	0.042	0.029	75.82	4.13	14.3	0-32.6	1	0	.	0	.	.
**2000**	26.5	11.6-41.3	1	0	.	0	.	.	73.5	58.7-88.4	1	0	.	0	.	.
**2001**	32.6	11.8-53.4	2	6.94	0.008	0.019	85.58	6.94	13.6	7.4-19.8	2	1.33	0.249	0.001	24.83	1.33
**2002**	40	18.5-61.5	1	0	.	0	.	.								
**2003**	36.5	0-99	2	81.97	0.000	0.201	98.78	81.97	31.4	18.6-44.1	1	0	.	0	.	.
**2004**	33.3	0-83	2	64.33	0.000	0.127	98.45	64.33	24.7	14.8-34.5	1	0	.	0	.	.
**2006**	0.8	0-3.1	1	0	.	0	.	.	11.9	3.6-20.1	1	0	.	0	.	.
**2007**	52.6	30.2-75.1	1	0	.	0	.	.								
**2008**	34.7	0-76.7	4	683.14	0.000	0.18	99.63	272.85	3.5	0-9.6	2	1.37	0.242	0.001	27.04	1.37
**2009**	42.5	23-62	2	5.14	0.023	0.016	80.56	5.14	13.3	3.4-23.3	1	0	.	0	.	.
**2010**	46.8	3.9-89.6	3	66.17	0.000	0.138	96.87	31.95	4.8	0-10.2	1	0	.	0	.	.
**2011**	29.1	6.5-51.8	4	72.49	0.000	0.046	93.15	14.6	38.2	21.9-54.6	1	0	.	0	.	.
**2012**	34.7	4.8-64.7	3	38.24	0.000	0.064	93.34	15.02	7.2	4.6-9.9	2	0.94	0.332	0	0	1
**2013**	8.6	2.5-14.8	1	0	.	0	.	.								
**2014**	40.6	0-96.1	3	467.81	0.000	0.239	99.61	253.63	4.7	0-10.2	2	4.53	0.033	0.001	77.91	4.53
**2015**	14.2	5.4-22.9	9	110.47	0.000	0.015	98.24	56.88	3	0.8-5.2	7	36.47	0.000	0	77.06	4.36
**2016**	16.3	1-31.6	3	12.93	0.002	0.014	84.78	6.57	0.7	0-2	2	0.13	0.719	0	0.11	1
**2017**	38.9	13.2-64.6	3	12.82	0.002	0.038	82.56	5.73	16.7	0-45.1	2	2.1	0.148	0.027	52.27	2.1
**2018**	32.2	11-53.4	4	109.32	0.000	0.043	94.85	19.4	15.6	0-35.3	3	26.51	0.000	0.027	90.67	10.71
**2019**	28.3	0-66.8	3	30.27	0.000	0.108	96.86	31.86	29.3	0-62.6	2	4.49	0.034	0.047	77.74	4.49
**2020**	1.2	0-2.8	1	0	.	0	.	.	1.2	0-2.8	1	0	.	0	.	.
**2021**	31.5	0-76.5	2	4.51	0.034	0.086	77.83	4.51	2.1	0-12.5	2	1.19	0.275	0.003	16.09	1.19
**2022**	19.7	13.2-26.1	3	0.76	0.683	0	0	1	8.5	1.4-15.6	1	0	.	0	.	.
**2023**	8.3	1.3-15.3	1	0	.	0	.	.	.	.	.	.	.	.	.	.
**2024**	14.6	1.7-27.5	2	3.62	0.057	0.006	72.36	3.62	.	.	.	.	.	.	.	.

%: prevalence rate; CI: confidence interval; P: P-value;

Table S6. Country-specific differences in positivity rate of HPV-16 and HPV-18 in oral cavity cancer patients

	HPV-16								HPV-18							
	Prevalence	95% CI	Studies	Q	P	Tau^2	I^2	H2	Prevalence	95% CI	Studies	Q	P	Tau^2	I^2	H2
**Australia**	53	45.8-60.1	3	0.29	0.864	0	0.01	1	40	0-82.9	1	0	.	0	.	.
**Brazil**	13.5	7.1-19.9	3	2.58	0.276	0.001	28.64	1.4	8.1	2.7-13.6	2	0.02	0.889	0	0.01	1
**Canada**	40	18.5-61.5	1	0	.	0	.	.	.	.	.	.	.	.	.	.
**China**	26.4	0-59.2	3	100.05	0	0.082	98.89	89.72	10.4	0-23.5	3	21.79	0	0.012	95.78	23.71
**Croatia**	19.2	4.1-34.4	1	0	.	0	.	.	.	.	.	.	.	.	.	.
**Czech Republic**	96.7	90.2-100	1	0	.	0	.	.	.	.	.	.	.	.	.	.
**Egypt**	33.3	24-42.6	1	0	.	0	.	.	.	.	.	.	.	.	.	.
**Germany**	52.3	31.4-73.3	3	6.78	0.034	0.023	68.93	3.22	21.1	7-41.6	2	4.73	0.03	0.017	78.88	4.73
**Greece**	2.6	0-6.7	2	1.59	0.208	0	36.98	1.59	1.9	0-5.5	1	0	.	0	.	.
**India**	25.8	16.6-34.9	17	266.85	0	0.031	97.48	39.62	14.5	7-22.1	14	98.9	0	0.016	98.28	58.29
**Indonesia**	1.3	0-3.8	1	0	.	0	.	.	15.4	7.4-23.4	1	0	.	0	.	.
**Iraq**	20	7.6-32.4	1	0	.	0	.	.	.	.	.	.	.	.	.	.
**Italy**	44.2	30.7-57.7	2	0.01	0.927	0	0	1	.	.	.	.	.	.	.	.
**Japan**	10.2	0.3-20.2	4	17.34	0.001	0.008	85.19	6.75	37.4	0-100	2	78.61	0	0.253	98.73	78.61
**Montenegro**	23.3	12.6-34	1	0	.	0	.	.	.	.	.	.	.	.	.	.
**Pakistan**	89.5	83.3-95.6	1	0	.	0	.	.	2.1	0-5	1	0	.	0	.	.
**Poland**	55.9	0-100	2	37.45	0	0.179	97.33	37.45	.	.	.	.	.	.	.	.
**Singapore**	41.8	31.6-51.9	1	0	.	0	.	.	47.3	37-57.5	1	0	.	0	.	.
**Slovenia**	4.8	0-10.2	1	0	.	0	.	.	.	.	.	.	.	.	.	.
**South Africa**	10.6	0-31.3	2	12.55	0	0.021	92.03	12.55	11.9	3.6-20.1	1	0	.	0	.	.
**Spain**	52.6	30.2-75.1	1	0	.	0	.	.	.	.	.	.	.	.	.	.
**Sweden**	8.2	3.1-13.3	1	0	.	0	.	.	.	.	.	.	.	.	.	.
**Taiwan**	27.5	3-51.9	4	39.33	0	0.058	99.29	141.27	5.8	4-7.6	3	2.88	0.237	0	24.89	1.33
**Thailand**	7.4	0-15.3	4	23.69	0	0.006	92.23	12.87	0.7	0.2-1.5	3	0.47	0.79	0	0.04	1
**USA**	23.3	0-56.5	2	6.34	0.012	0.049	84.23	6.34	14.3	0-32.6	1	0	.	0	.	.
**Venezuela**	44	30.2-57.8	1	0	.	0	.	.	10	1.7-18.3	1	0	.	0	.	.

CI: confidence interval

Table S7. Differences in HPV-16 and HPV-18 positivity rates across various patients' groups

	HPV-16			HPV-18		
	**Prevalence (%)**	**95% Confidence Interval**	**Number of Studies**	**Prevalence (%)**	**95% Confidence Interval**	**Number of Studies**
**Gender**						
Female	23.5	11.8-35.3	6	19.9	11.1-28.8	4
Male	23.5	13-33.9	6	20.5	7.1-33.9	4
**Histological Type**						
MD	26.8	18.2-35.5	6	39.1	11.7-66.5	4
PD	36.5	23.3-49.7	6	31.4	8.3-54.5	4
VC	40	15.2-64.8	1	40	15.2-64.8	1
WD	28	19.9-36.2	6	26.1	8.4-43.8	4
**P16**						
Negative	11.3	0-24.6	2	11.3	0-24.6	2
Positive	23.8	0-71.5	2	23.8	0-71.5	2
**Age**						
40-60	28	21.4-34.6	14	24.7	14.4-34.9	9
60-70	29.1	19.5-38.8	4	42.7	27.5-57.8	2
<40	42.2	22.2-62.3	5	26.8	7.5-46.1	4
>70	14.7	0-37.4	2	50	23.8-76.2	1
**Clinical TNM**						
I	96.9	88.3-100	1	3.1	0-11.7	1
II	83.3	53.5-100	1	7.1	0-26.2	1
III	15.4	0-35	1	38.5	12-64.9	1
IV	2.9	0-11	1	2.9	0-11	1
**Site**						
Alveolus	9.1	0-26.1	1	4.2	0-15.5	1
Buccal Mucosa	31.2	22.4-40	4	28.8	9.6-48.1	4
Floor of Mouth	50	25.5-74.5	2	33.3	0-71.1	1
Gingiva	33.3	0-71	1	7.1	0-26.2	1
Lip	10	0-28.6	2	10	0-28.6	2

Lower Alveolus	60	29.6-90.4		40	9.6-70.4	1
Lower Lip	14.3	1.3-27.2	1			
Mandibular Gingiva	25	3.8-46.2	1	31.2	8.5-54	1
Maxillary Gingiva	7.7	0-22.2	1	15.4	0-35	1
Oral Tongue	31.8	16-47.5	8	32.4	8.2-56.6	4
**Pathological TNM**						
I	26.9	0-69.7	2	71.4	38-100	1
I-II	17.4	1.9-32.9	1	8.7	0-20.2	1
II	28.3	21-35.7	2	61.1	38.6-83.6	1
III	20.8	0-54.8	2	42.9	6.2-79.5	1
III-IV	24.1	15.1-33.1	1	10.3	3.9-16.7	1
IV	16.7	0-37.8	1	50	21.7-78.3	1
**Pathological N**						
N+	15.8	0-32.2	1	15.8	0-32.2	1
N0	32.4	3.5-61.4	2	50	21.7-78.3	1
N1	18.8	5.2-32.3	1	-	-	-
N2	32.5	22-42.9	1	-	-	-
N3	53.3	28.1-78.6	1	-	-	-
**Smoking**						
Current	18.3	10.6-25.9	2	15.8	0-32.2	1
Never	54.6	33.9-75.3	2	50	21.7-78.3	1
**Alcohol**						
Ever	22.2	0-49.4	1	22.2	0-49.4	1
Never	31.8	12.4-51.3	1	31.8	12.4-51.3	1
**Pathological T**						
T1	9.9	0.4-19.5	3	71.4	38-100	1
T2	21.8	12.4-31.3	4	57.1	36-78.3	1
T3	28.5	16.8-40.3	4	54.5	25.1-84	1
T4	17.7	1.4-34	4	28.6	0-62	1
